# The *dlx5a/dlx6a* Genes Play Essential Roles in the Early Development of Zebrafish Median Fin and Pectoral Structures

**DOI:** 10.1371/journal.pone.0098505

**Published:** 2014-05-23

**Authors:** Églantine Heude, Sarah Shaikho, Marc Ekker

**Affiliations:** Centre for Advanced Research in Environmental Genomics, Department of Biology, University of Ottawa, Ottawa, Ontario, Canada; Institute of Molecular and Cell Biology, Singapore

## Abstract

The *Dlx5* and *Dlx6* genes encode homeodomain transcription factors essential for the proper development of limbs in mammalian species. However, the role of their teleost counterparts in fin development has received little attention. Here, we show that *dlx5a* is an early marker of apical ectodermal cells of the pectoral fin buds and of the median fin fold, but also of cleithrum precursor cells during pectoral girdle development. We propose that early median fin fold establishment results from the medial convergence of *dlx5a*-expressing cells at the lateral edges of the neural keel. Expression analysis also shows involvement of *dlx5a* during appendage skeletogenesis. Using morpholino-mediated knock down, we demonstrate that disrupted *dlx5a/6a* function results in pectoral fin agenesis associated with misexpression of *bmp4*, *fgf8a, and1* and *msx* genes. In contrast, the median fin fold presents defects in mesenchymal cell migration and actinotrichia formation, whereas the initial specification seems to occur normally. Our results demonstrate that the *dlx5a/6a* genes are essential for the induction of pectoral fin outgrowth, but are not required during median fin fold specification. The *dlx5a/6a* knock down also causes a failure of cleithrum formation associated with a drastic loss of *runx2b* and *col10a1* expression. The data indicate distinct requirements for *dlx5a/6a* during median and pectoral fin development suggesting that initiation of unpaired and paired fin formation are not directed through the same molecular mechanisms. Our results refocus arguments on the mechanistic basis of paired appendage genesis during vertebrate evolution.

## Introduction

In vertebrates, appendages (limbs, wings and fins) show major structural and functional differences, but they present remarkable similarities in their developmental mechanisms [1]. Genes known to play a critical role in the initiation, growth, and patterning of tetrapod limbs (e.g. *Tbx, Hox, Fgf, Bmp and Shh*) are expressed in comparable spatiotemporal domain in fins [2]–[5] and share similar functions [5]–[11]. Particularly, the tetrapod *Dlx5*/*Dlx6* and teleost *dlx5a/dlx6a* genes are expressed in the apical ectodermal ridge (AER) of the developing limbs in mice [12]–[15] and in the pectoral fin fold (PFF) and median fin fold (MFF) giving rise respectively to paired and unpaired fins in zebrafish [16]–[19]. At early stage of fin morphogenesis, teleosts present an AER structurally homologous to the AER of tetrapods [1], [20]. Later, the AER transitions into an elongated pectoral fin fold [20]. AER and fin fold structures have been demonstrated to be essential signaling centers during appendage specification and outgrowth in vertebrates [7], [17], [20]–[22]. Despite the fact that tetrapod studies have demonstrated the central role of *Dlx5/6* genes in limb formation, the implication of *dlx5a/6a* genes in teleost fin development has been little analyzed beyond examination of expression patterns.

*Dlx* genes code for an evolutionary conserved group of homeodomain transcription factors, related in sequence to the *Drosophila distalless* gene (*dll*) essential for distal appendage patterning in insects [23]. The *Dlx* genes have arisen from the ancestral *dll* gene as a result of gene duplication events [24]. In tetrapods, the *Dlx* family consist of six genes organized into the *Dlx1/2*, *Dlx3/4* and *Dlx5/6* bigene clusters. In zebrafish, eight *dlx* genes have been reported among which six (*dlx1a/2a, dlx3b/dlx4b, dlx5a/6a*) are arranged on chromosomes similarly to their tetrapod counterparts [24]–[27]. Expression and functional analyses of *Dlx5/6* have demonstrated their key roles in the development of the nervous system, of craniofacial structures, of endochondral bones and of appendages [13], [14], [28]–[38]. Simultaneous inactivation of *Dlx5* and *Dlx6* in the mouse results in a limb phenotype similar to that observed in patients affected with split-hand split-foot malformation type I (SHFM-I) [14], [34], [37]. Altered limb development in *Dlx5/6* null mice is associated with loss of *Bmp4*, *Fgf8* and *Msx2* expression in the medial part of the AER [14], [15]. The data indicate that the *Dlx5/6* genes have a central role in vertebrate appendage formation. It was therefore of interest to examine the function of *dlx5a/6a* during the development of paired and unpaired fins.

Here, we show that *dlx5a/6a* genes are required for the initiation of pectoral fin outgrowth and for median fin fold morphogenesis. Our results suggest distinct requirements for *dlx5a/6a* genes in paired and unpaired fin development. Moreover, the analyses demonstrate that *dlx5a/6a* are implicated in cleithrum formation and suggest that *dlx5a* is involved in fin skeletogenesis.

## Materials and Methods

### Ethical statement

All experiments were performed according to the guidelines of the Canadian Council on Animal Care and were approved by the University of Ottawa animal care committee (institutional licence #BL 235). All efforts were made to minimize suffering; manipulations on adult animals were performed with the anaesthetic drug tricaine mesylate (ethyl 3-aminobenzoate methanesulfonate; Sigma-Aldrich, Oakville, ON, Canada). Embryos were killed with an overdose of the latter drug.

### Animal maintenance

Zebrafish and their embryos were maintained at 28.5°C according to methods described in [39]. Wild-type adult zebrafish were kept and bred in circulating fish water at 28.5°C with a controlled 14-h light cycle. Embryos were collected at the one-cell-stage. A Narishige IM300 microinjector was used for microinjection. Wild-type, controls, and injected embryos were raised at similar densities in embryo medium in a 28.5°C incubator. Embryos were treated with 0.0015% 1-phenyl 2-thiourea (PTU) to inhibit melanogenesis.

### Morpholino-mediated knock down and rescue experiment constructs

For morpholino-mediated knock down, we injected or co-injected in 1 cell-stage embryos, 1 nl of *dlx5a* and/or *dlx6a* morpholinos at a concentration of either 0.4 mM or 0.8 mM. The choice of morpholino concentrations has been determined performing injection or co-injection of *dlx5a* and/or *dlx6a* in a range from 0.2 to 1.6 mM. Injection of 0.2 mM MOs did not lead to any obvious phenotype whereas injection of 1.6 mM MOs was lethal after a few hours post-injection. The 5′-untranslated region of each *dlx* gene was used to design translation-blocking antisense MOs against each *dlx* transcript. The following translation-blocking MOs were obtained from Gene Tools (LLC, Philomath, OR, USA): *dlx5a* MO 5′-TCCTTCTGTCGAATACTCCAGTCAT-3′; *dlx6a* MO 5′-TGGTCATCATCAAATTTTCTGCTTT-3′.

The following splice-blocking MOs that target exon 2 excision were also designed to confirm the phenotype obtained using the translation-blocking morpholinos: *dlx5a* e2i2 MO 5′-TATTCCAGGAAATTGTGCGAACCTG-3′; *dlx6a* e2i2 MO 5′-AAATGAGTTCACATCTCACCTGCGT-3′ (from Gene Tools, LLC). Although the efficacy of the *dlx5a* e2i2 morpholino was deemed to be insufficient by Talbot *et al.* [38], in our hands, injection of 0.4 mM *dlx5a* e2i2 MO caused a 57% decrease in the levels of *dlx5a* transcripts. We observed comparable fin phenotypes injecting either *dlx* translation- or splice-blocking morpholinos (data not shown).

As controls, we injected water or 1.6 mM of Standard Control MO (Gene Tools) that targets a human beta-globin intron mutation that causes beta-thalessemia. (Gene Tools 5′-CCTCTTACCTCAGTTACAATTTATA-3′).

To ensure specificity of the morpholinos, rescue of the resulting morphant phenotypes was performed by co-injecting the corresponding *dlx5a/6a* mRNAs mutagenized on the MO target site (*dlx5a* MO binding site T(ATG)ACTGGAGTATTCGACAGAAGGA, mut*dlx5a* sequence **C**(ATG)AC**G**GG**T**GT**T**TT**T**GA**T**AGGAGGA; *dlx6a* MO binding site AAAGCAGAAAATTTG(ATG)ATGACCA, mut*dlx6a* sequence A**TT**GCA**A**A**T**AAT**A**TG(ATG)ATGAC**C**A). Vectors were linearized with *Not*I and mRNAs were synthesized using the SP6 mMessage mMachine kit (Ambion).

### In situ hybridization

*In situ* hybridization on whole-mount embryos and cryostat sections were performed as previously described [40], [41].

For whole-mount *in situ* hybridization, embryos from 12 to 96 hpf (n>100 for each experimental groups and markers) were fixed in 4% paraformaldehyde (PFA, Millipore) in phosphate buffer saline 1× (PBS, Amresco) overnight at 4°C, dehydrated in methanol, and stored in 100% methanol at −20°C.

For *in situ* hybridization on cryostat sections, larvae (n = 16) were fixed in 4% PFA in PBS overnight at 4°C, washed in PBS and equilibrated in 30% sucrose in PBS overnight at 4°C. The samples were then embedded and frozen in O.C.T compound (Sakura Finetek) and sectioned at 10 µm. After *in situ* hybridization, sections were mounted with coverslips and Aqua Poly/Mount (Polysciences) before imaging.

The antisense mRNA probes were labeled with digoxygenin-11-UTP (Roche) and synthesized from cDNA clones: *dlx5a* [42], *dlx6a* [29], *bmp4* [43], *fgf8a* [44], *msxB* [45], *msxC* [45], *and1* [46], *runx2b* [43], *col10a1* [47]. NBT/BCIP (Roche) was used as alkaline phosphatase substrate.

### Whole-mount TUNEL assay

The whole-mount TUNEL assay was performed on 24 hpf embryos (control embryos n = 62, *dlx5a/dlx6a* morphants n = 98) with the Apoptag peroxidase in situ apoptosis detection kit (Millipore) following the modifications described in [48].

### BrdU assay

Dechorionated embryos at 23 hpf were placed in embryo medium containing 10 mM BrdU (5-bromo-2′-deoxy-uridine, Roche) with 1% DMSO for 1 h at 28.5°C (control embryos n = 13, *dlx5a/dlx6a* morphants n = 28). Embryos were killed with an overdose of tricaine mesylate and fixed in 4% PFA in PBS overnight at 4°C, washed in PBDT (PBS DMSO 1% Tween 0.1%), dehydrated in methanol and stored in methanol 100% at −20°C. Then, the samples were rehydrated in a graded methanol-PBDT series, treated with proteinase K (10 µg/ml) for 20 minutes, post-fixed in PFA 4% for 20 minutes and incubated in 2N HCl for 1 h at room temperature.

Proliferating cells were immunodetected in wholemount embryos using a 1∶100 dilution of the mouse anti-BrdU monoclonal antibody (Sigma), a 1∶200 dilution of secondary anti-mouse HRP-conjugated antibody (Jackson Immuno), and revealed with DAB chromogenic substrate (Abcam).

### Histological picrosirius red staining

48 hpf embryos (n = 119) were fixed overnight in 4% PFA at 4°C and washed in PBST. The embryos were incubated for 1 h in a 0.2% solution of sirius red (direct red 80, Sigma-aldrich) dissolved in saturated picric acid (Sigma-aldrich). The staining was followed by washes in distilled water. Once the water ran clear and without any red color, embryos were sequentially dehydrated into glycerol/PBS solutions and stored in 100% glycerol.

## Results

### *dlx5a/dlx6a genes* are early markers of apical ectodermal cells in developing paired and unpaired fins

**Figure 1:**
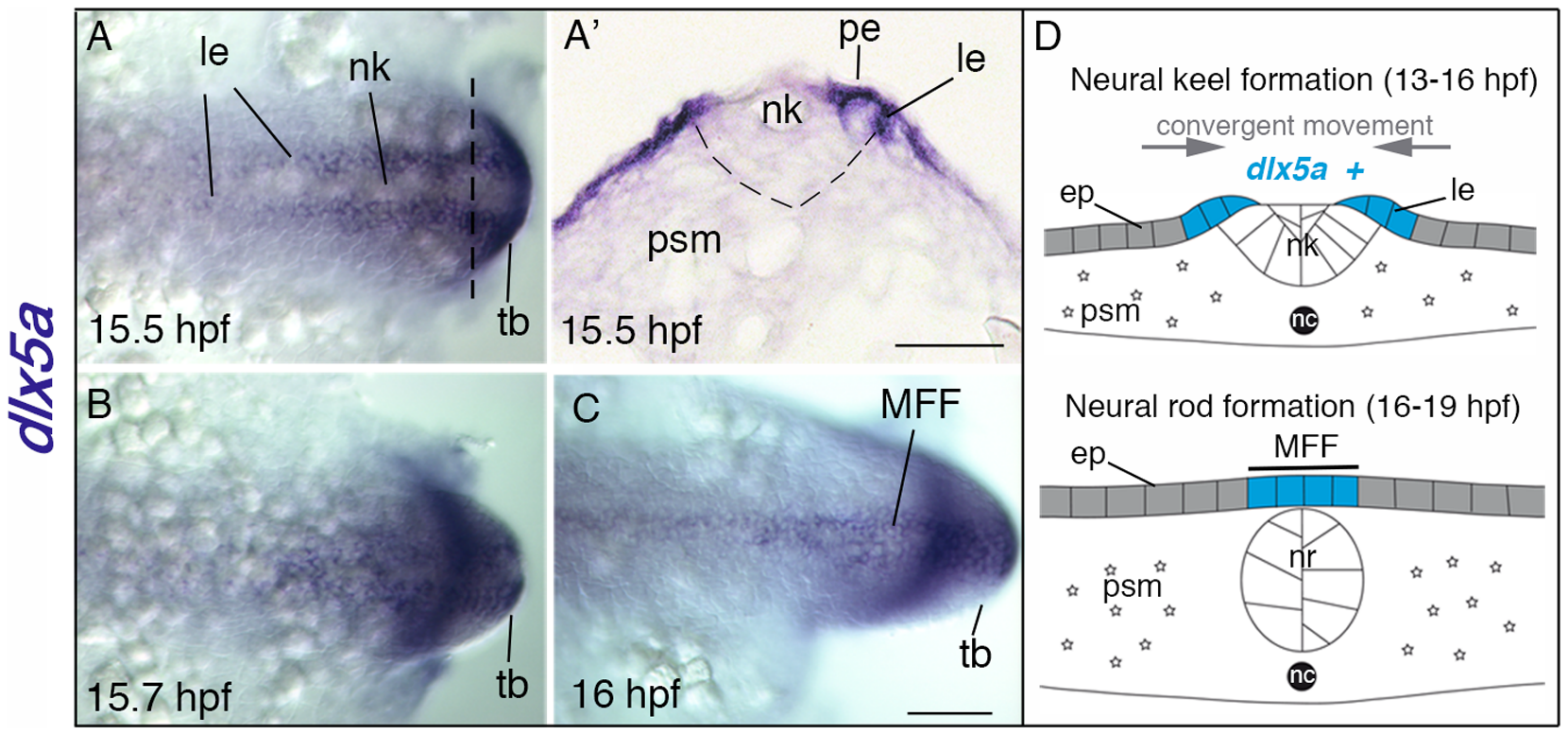
Expression of *dlx5a* during early specification of median fin fold ectodermal cells. (A–C) *In situ* hybridization for *dlx5a* in zebrafish embryos from 15.5 hpf to 16 hpf: dorsal view of the posterior axis (A, C) and coronal section (A′) at the level indicated by the dashed line in (A). At 15.5 hpf, *dlx5a* is expressed in ectodermal cells at the lateral edges (le) of the neural keel (nk) underlying the periderm (pe) (A, A′) (the dashed line in A′ delineates the neural keel). From 15.5 hpf to 16 hpf, ectodermal cells expressing *dlx5a* follow a dynamic convergent movement to form the presumptive median fin fold (MFF) at the dorsal midline of the embryo (B–C). (D) Schematic representation of the zebrafish dorsal cellular movement implicating *dlx5a* based on A–C. The convergent movement produced by the establishment of the neural rod (nr) (16–19 hpf) leads to the fusion of the two lateral edges at the midline into the presumptive MFF expressing *dlx5a*. ep, epidermis; nc, notochord; psm, presomitic mesoderm; tb, tail bud. Scale bars shown in C for A–C and in A′ 50 µm.

Expression of the zebrafish *dlx* genes has been previously examined [16], [19], [38], [49] including in the developing appendages. Even before the median fin fold becomes distinguishable, *dlx5a* transcripts are expressed in ectodermal cells underlying the periderm at the lateral edges of the neural keel at 15.5 hpf (Fig. 1 A, A′). From 15.5 hpf to 16 hpf, *dlx5a*-expressing cells follow a dynamic convergent movement toward the dorsal midline to form the presumptive median fin fold (MFF) (Fig. 1 A–D), the anterior expression limit corresponding to the MFF domain around the 8^th^ somite [17]. Then, *dlx5a* expression is limited to MFF ectodermal cells at 24 hpf and 48 hpf (Fig. S1), and gradually decreases until 72 hpf when transcripts are hardly detectable (data not shown). Expression of *dlx6a* mirrors that of *dlx5a* except that transcripts seem to be present at lower levels (Fig. S2 B, D), a difference that was observed by us and others throughout the embryo using a variety of probes for this gene [18], [19], [29].

**Figure 2:**
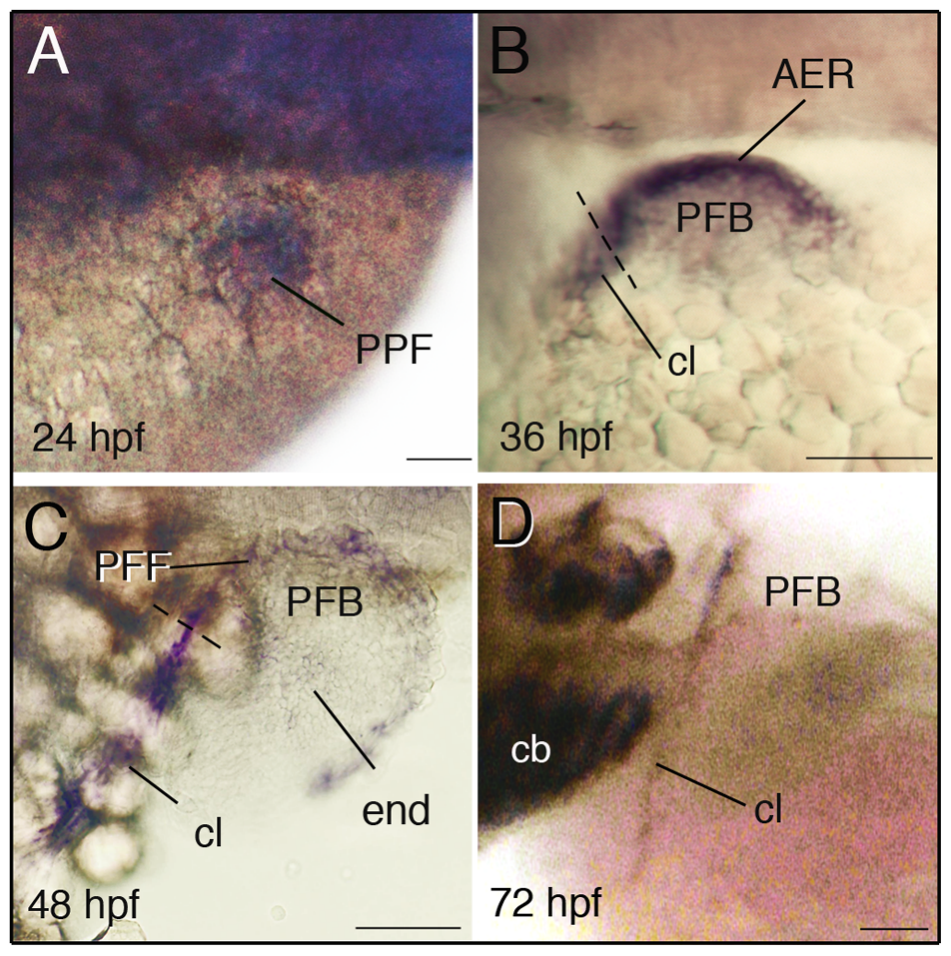
Expression of *dlx5a* during zebrafish pectoral development. Whole mount *in situ* hybridization for *dlx5a* in the pectoral region of zebrafish embryos from 24 hpf to 72 hpf. During pectoral fin formation, *dlx5a* is expressed in apical ectodermal cells of the presumptive pectoral fin bud (PPF) at 24 hpf (A) and in the apical ectodermal ridge (AER) of the early pectoral fin bud (PFB) at 36 hpf (B). At 48 hpf, *dlx5a* expression is detected in the pectoral fin fold (PFF) and weak expression is observed in endochondral cells (end) of the PFB. Moreover, the transcripts are detected in the developing cleithrum (cl) from 36 hpf to 72 hpf (B–D). The dashed lines in B–C indicate the limit between the AER/PFF structures and cleithrum precursor cells. Note the absence of *dlx5a* expression in the PFF at 72 hpf (D). Scale bars 50 µm.

At the pectoral level, *dlx5a* transcripts are first detected at 24 hpf in apical ectodermal cells of the presumptive pectoral fin bud (Fig. 2 A). At 36 hpf, *dlx5a* is highly expressed in the AER of the developing pectoral fin buds (Fig. 2 B). From 36 hpf to 48 hpf, the AER develops into the pectoral fin fold (PFF) in zebrafish embryos [20]. During PFF establishment, *dlx5a* expression is maintained in apical ectodermal cells until 48 hpf (Fig. 2 C). Transcript levels progressively decrease in the PFF from 48 hpf to 72 hpf (Fig. 2 D). Similar observations were made for *dlx6a*, although transcript levels appear to be weaker (Fig. S2 A, C).

Transcripts of *dlx5a* are also expressed in the developing cleithrum at the base of the pectoral fin bud which extends the AER/PFF *dlx5a*-positive domain ventro-laterally from 36 to 72 hpf (Fig. 2 B–D). The cleithrum is one of the major bones of the pectoral girdle which supports the pectoral fins in bony fish. Weak *dlx5a* expression is also detected in the endochondral disc of the pectoral fin bud at 48 hpf and 54 hpf (Fig. 2 C and data not shown). These observations show that *dlx5a*, and to a lesser extent *dlx6a*, are early markers of ectodermal cells giving rise to PFF and MFF structures and of precursor cells of the developing cleithrum and pectoral endochondral disc.

### Knock down of *dlx5a/6a* leads to severe appendage defects

**Figure 3:**
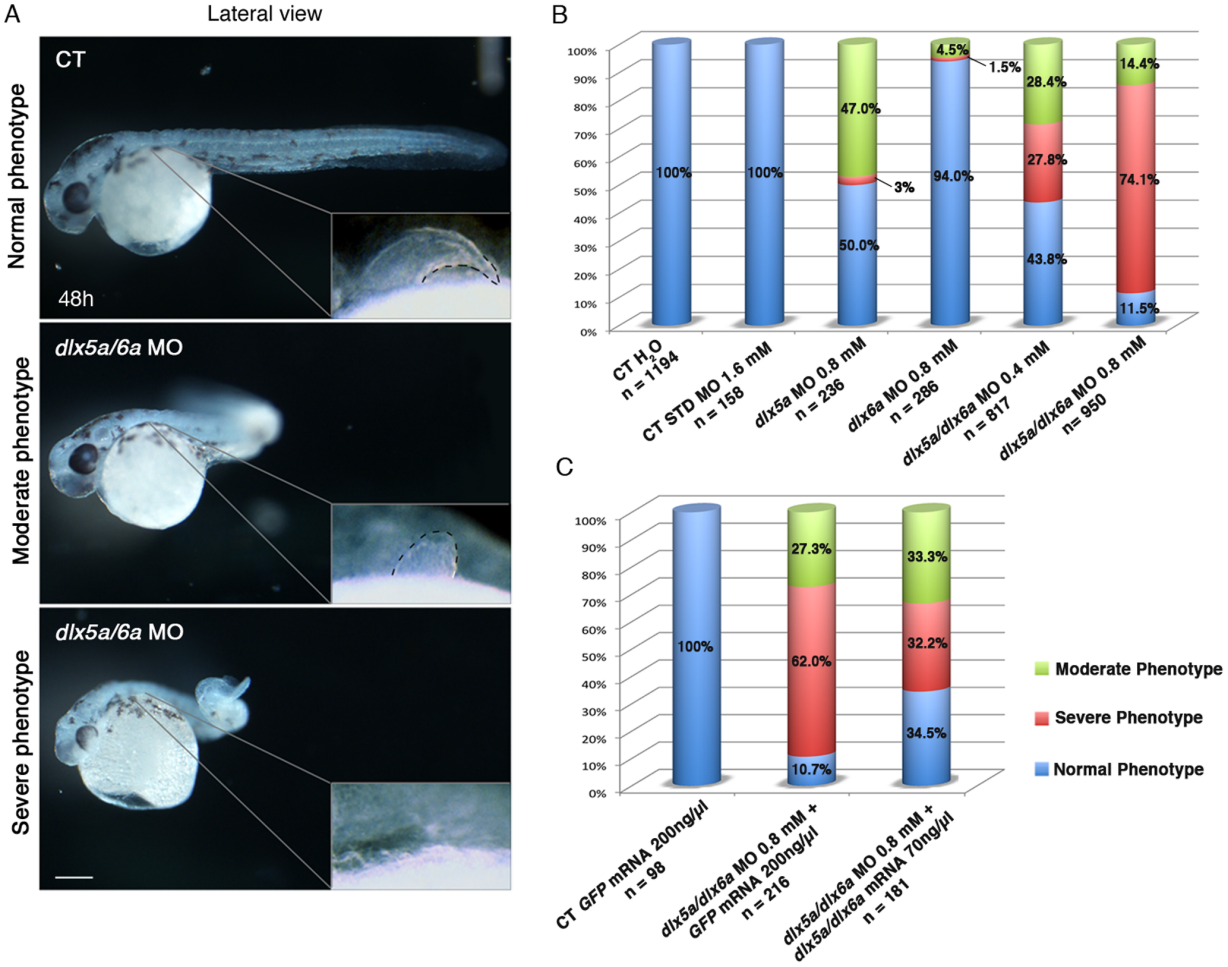
Phenotypes obtained with different *dlx5a/dlx6a* morpholinos and mRNA treatments. (A) Phenotypes observed in *dlx5a/6a* morphant embryos. Lateral view of control (CT) and *dlx5a/6a* morphant embryos at 48 hpf. The *dlx5a/6a* gene knock down results in moderate “curved tail” and severe “ curly tail” phenotypes compared to controls. The moderate and severe phenotypes are associated with hypoplasia and agenesis of pectoral fin bud respectively as shown in the pectoral region magnifications. Scale bar for all panels 100 µm. (B, C) The graphics show the percentages of normal (blue bars), moderate (green bars) and severe (red bars) phenotypes obtained at 48 hpf following injection of different *dlx5a/dlx6a* MOs and *dlx5a/dlx6a* mRNAs. For each treatment, the number (n) of specimens analyzed is indicated and each experiment was performed at least 3 times. The B graph shows the following treatments: control embryos injected with H_2_O; control embryos injected with a control MO (1.6 mM); single morphants injected with either *dlx5a* or *dlx6a* MOs (0.8 mM); double morphants co-injected with *dlx5a* and *dlx6a* MOs at two different concentrations (0.4 mM or 0.8 mM each). The C graph shows rescue experiments: control embryos injected with *GFP* mRNA (200 ng/ µl); control embryos co-injected with *dlx5a/6a* morpholinos (0.8 mM each) and *GFP* mRNA (200 ng/ µl) and embryos co-injected with *dlx5a/6a* morpholinos (0.8 mM each) and *dlx5a/dlx6a* mRNAs (70 ng/ µl each).

To analyze the implication of *dlx5a/6a* genes in appendage development, we performed *dlx5a* and *dlx6a* knock down in zebrafish embryos using morpholinos (MO). We performed micro-injections at the 1 cell-stage with one morpholino or co-injection of two *dlx* morpholinos at two different concentrations (0.4 mM and 0.8 mM). Embryos injected with translation-blocking MOs against *dlx5a* and/or *dlx6a* exhibited characteristic and reproducible moderate to severe phenotypes compared to control embryos (Fig. 3 A). When observed at 48 hpf, a moderate phenotype is defined as presence of a “curved tail” and hypoplastic pectoral fins whereas the severe phenotype corresponds to a “curly tail” associated with agenesis of pectoral fins. Moreover, embryos with a severe phenotype are generally smaller in size when compared to controls and display craniofacial malformations (Fig. 3 C).

In single *dlx5a* morphants (0.8 mM), 118/236 (50%) of the embryos exhibit the normal phenotype whereas 111/236 (47%) show moderate and 7/236 (3%) display severe phenotypes (Fig. 3 B). Knock down of *dlx6a* (0.8 mM) leads to mostly normal phenotype (269/286) and we obtain a low proportion of moderate (13/286) and severe (4/286) phenotypes. The co-injection of *dlx5a* and *dlx6* MOs (0.4 mM each) increases the rate of moderate (232/817; 28%) and severe (227/817; 28%) phenotypes. When we increase the concentration of the injected MOs (0.8 mM each), the double knock down results mainly in severe phenotype (704/950) and low rate of moderate (137/950; 14%) and normal (109/950; 12%) phenotypes. Co-injection of splice-blocking *dlx5a/dlx6a* MOs was performed (n = 627) and confirmed the results obtained using translation-blocking MOs. The phenotypes of single *dlx* morphants compared to double *dlx5a/6a* morphant embryos underlie the potentially redundant function of the *Dlx* paralogs in vertebrates [13], [14], [50]–[52]. Moreover, the results show that the phenotypes observed in double morphants are dose-dependent. When we doubled the concentration of the MOs from 0.4 mM to 0.8 mM each, the rate of severe phenotype also increases from 27.8% to 74.1%. Based on the above observations, we performed subsequent experiments by injecting *dlx5a + dlx6a* MOs at 0.8 mM and we considered the severe phenotype embryos as specimens in which the *dlx5a/dlx6a* knock down was more efficient. Thus, the *dlx5a/dlx6a* morphants analyzed in the study are embryos presenting a severe phenotype.

We performed *dlx5a/dlx6a* mRNA rescue experiments to test the specificity of the phenotypes obtained with the *dlx5a* and *dlx6a* MOs (Fig. 3 C). We mutagenized 5′-UTR sequences in the *dlx* mRNAs that were co-injected with the *dlx5a/6a* MOs to prevent MO binding. The results show that co-injection of *dlx5a/6a* MOs and *dlx5a/6a* mRNAs (70 ng/ µl each) increases three times the proportion of normal phenotype and decrease almost twice the number of severe phenotype embryos compared to the *dlx5a/6a* double morphants co-injected with GFP mRNA (200 ng/ µl) (Fig. 3 C). The experiments suggest that these phenotypes are specific to the *dlx5a/6a* knock down. The phenotypic aspects, including craniofacial malformations, pectoral fin and MFF defects, not fully rescued in the embryos with mild and severe phenotype, can be explained by the aberrant ubiquitous *dlx5a/6a* overexpression which leads to mild developmental defects when injected alone in the embryo (data not shown).

### The *dlx5a/6a* genes are required for the induction of pectoral fin outgrowth and cleithrum differentiation

To better understand the effects of *dlx5a/6a* knock down on zebrafish fin development, we analyzed the expression of different markers known to be involved in appendage specification and morphogenesis, including some that have been shown to be affected in *Dlx5/6^−/−^* mouse embryos [14], [15].

**Figure 4:**
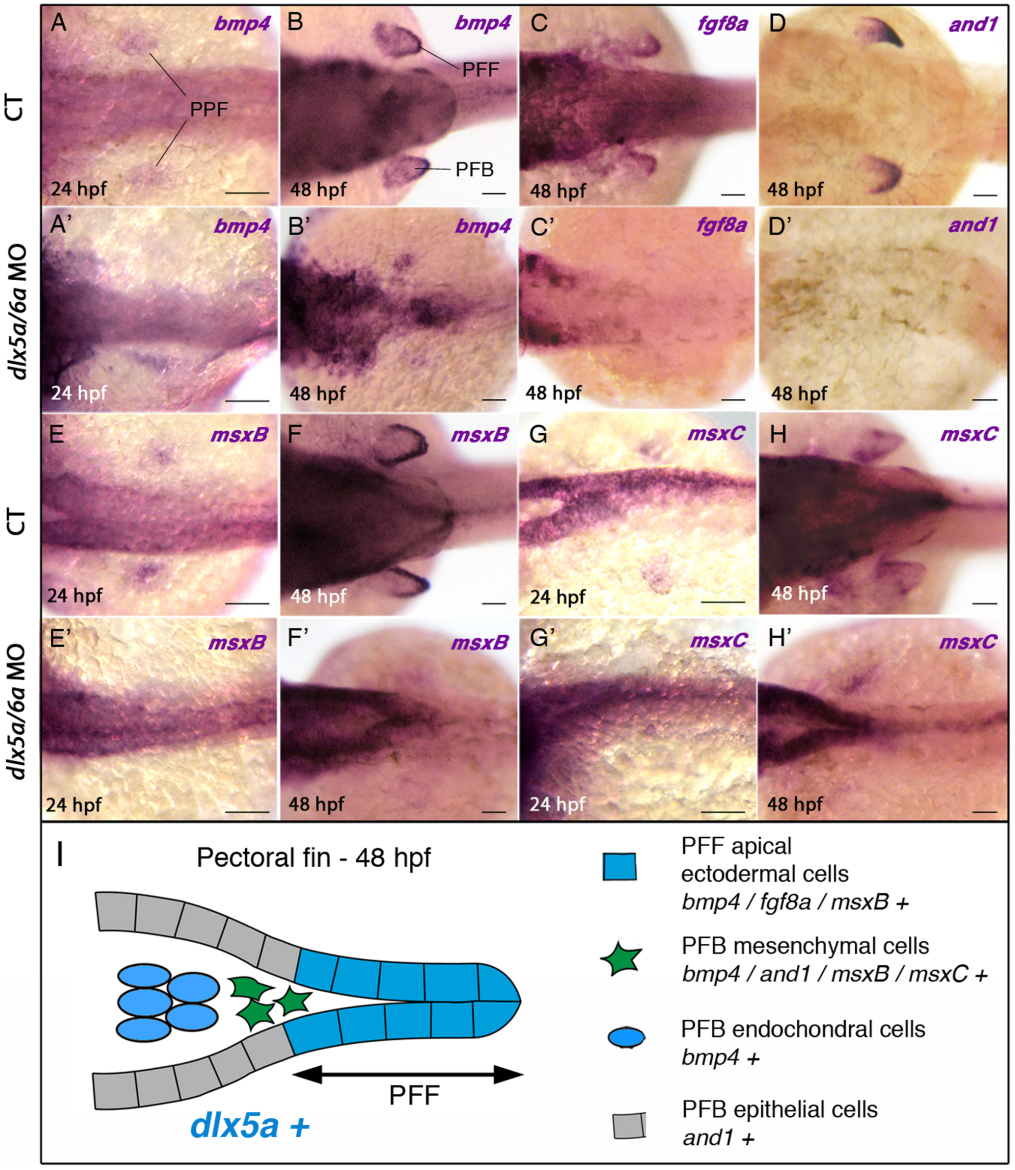
Impaired expression of *bmp4*, *fgf8a, and1* and *msx* genes in the pectoral fin region of *dlx5a/6a* morphants. Whole mount *in situ* hybridization for *bmp4* (A, A′–B, B′), fg8a (C, C′), *and1* (D, D′), *msxB* (E, E′–F, F′) and *msxC* (G, G′–H–H′) at 24 and 48 hpf in dorsal views of control (A–H) and *dlx5a/6a* morphant (A′–H′) embryos. At 24 hpf, *bmp4*, *msxB* and *msxC* genes are expressed in apical ectodermal cells of the presumptive pectoral fin bud (PPF) in control embryos (A, E, G). In *dlx5a/6a* morphants, *bmp4* expression is lost or altered in the presumptive pectoral fin bud (A′) and the *msxB* and *msxC* transcripts are hardly detectable (E′, G′). In 48 hpf control embryos, *bmp4* is expressed in the pectoral fin fold (PFF), the underlying mesenchyme and in mesodermal cells of the pectoral fin bud (PFB) (B). At the equivalent stage, *fgf8a* is detected in PFF ectodermal cells (C), and *and1* expression is observed in the distal mesenchyme and in epithelial cells of the PFB but not in the PFF (D). The *msxB* gene is expressed in the PFF and the underlying mesenchyme (F), and *msxC* is detected in the mesenchymal cells but not in the PFF (H). In contrast to what is observed in controls at 48 hpf, *dlx5a/6a* morphants show a marked decrease or loss of expression of the PFB markers associated with pectoral fin agenesis (B′–D′, F′, H′). (I) Schematic representation of the pectoral fin bud at 48 hpf summarizing the expression of *dlx5a* and the analyzed PFB markers in their corresponding cellular types. Scale bars 50 µm.

First, we examined expression of *bmp4*, *msxB* and *msxC* genes that are expressed in the presumptive pectoral fin bud of 24 hpf CT embryos, at a position where pectoral fin buds will appear a few hours later (Fig. 4 A, E, G). In 24 hpf *dlx5a/6a* morphants, we observed that the expression of the analyzed genes is lost in the presumptive pectoral fin bud during pectoral fin specification (Fig. 4 A′, E′, G′).

We then analyzed the expression of markers that are involved during pectoral fin morphogenesis. At 48 hpf, *bmp4* transcripts are found in the pectoral fin fold (PFF), the underlying mesenchyme and in the whole pectoral fin mesoderm (Fig. 4 B), whereas *fgf8a* expression is only detected in the PFF (Fig. 4 C). At the same stage, *and1* is expressed in epithelial cells of the pectoral fin bud, and in distal mesenchymal cells invading the fold [46] (Fig. 4 D). Moreover, Expression of *msxB* is limited to PFF cells and to the adjacent mesenchymal cells whereas *msxC* is only detected in mesenchymal cells underlying the PFF [45] (Fig. 4 F, H). In contrast, 48 hpf *dlx5a/6a* morphants exhibit a severe decrease or complete loss of expression of the analyzed genes in the pectoral region at (Fig. 4 F′, H′).

These results show that the *dlx5a/6a* knock down leads to severely impaired or abolished expression of genes implicated in early vertebrate appendage development, a phenotype characterized by agenesis of pectoral fin buds.

**Figure 5:**
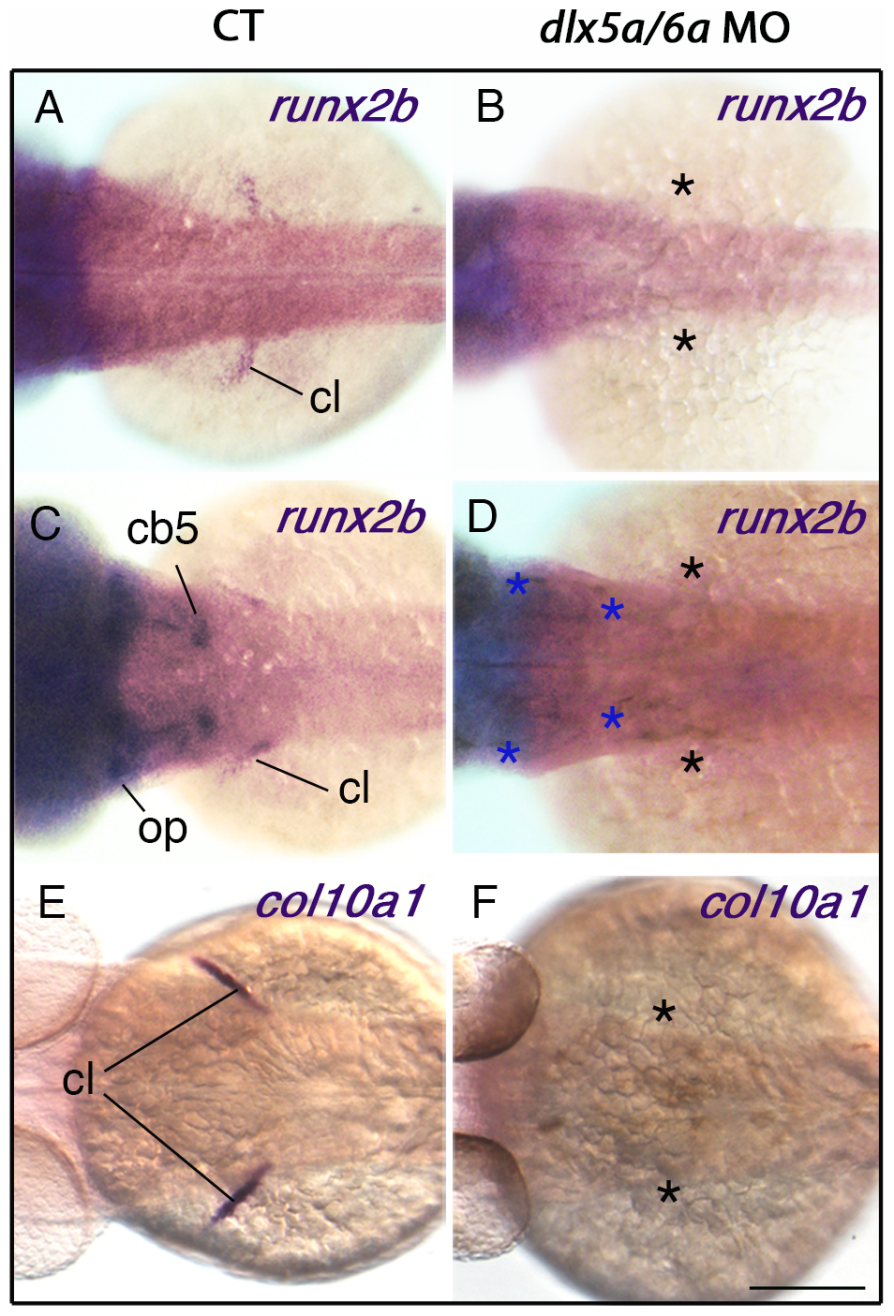
Knock down of *dlx5a/6a* leads to a defect of cleithrum differentiation. Dorsal views of whole mount *in situ* hybridization for *runx2b* and *col10a1* on control (A, C, E) and *dlx5a/6a* morphant (B, D, F) embryos at 36 hpf (A–B) and 48 hpf (C–F). In controls at 36 hpf, *runx2b* is expressed in precursors cells of the cleithrum (cl) (A) whereas expression is absent in the pectoral region of *dlx5a/6a* morphants (B, black asterisks). At 48 hpf, expression of *runx2b* and *col10a1* is detected in differentiating osteoblasts of the cleithrum which supports the pectoral fin bud (C, E). The *runx2b* transcripts are also observed at the craniofacial level in the opercular (op) and ceratobranchial 5 (cb5) bone precursors (C). In contrast, *dlx5a/6a* morphants show a drastic loss of *runx2b* and *col10a1* expression in the pectoral region (D, F, black asterisks) and of *runx2b* expression at the craniofacial level (D, blue asterisks). Scale bar shown in F for all panels 100 µm.

As previously mentioned, *dlx5a* is highly expressed in the developing cleithrum of wild-type embryos from 36 hpf to 72 hpf (Fig. 2 B–D). The cleithrum is a dermal bone [1] located at the base of the pectoral fin buds and is the first bone that mineralizes at the axial level during early zebrafish development [1], [53]. In 36 hpf control embryos, *dlx5a* expression in the cleithrum is associated with *runx2b* expression (Fig. 5 A), an early/intermediate stage marker of osteoblast differentiation. At 48 hpf, *runx2b* expression is maintained in the cleithrum and is also detected in the opercular and ceratobranchial-5 bones at the craniofacial level (Fig. 5 C). At the same stage, cells of the developing cleithrum highly express the *col10a1* gene (Fig. 5 E), an intermediate/late stage marker of osteoblast differentiation in zebrafish [47], [53], [54]. Interestingly, we show that *dlx5a/6a* knock down leads to a drastic loss of *runx2b* and *col10a1* expression in the cleithrum at 36 hpf and 48 hpf (Fig. 5 B–F), even in embryos presenting a moderate phenotype with hypoplastic pectoral fin buds (data not shown). At 48 hpf, loss of *runx2b* expression in *dlx5a/6a* morphants is also observed in the opercular and ceratobranchial-5 bone precursors (Fig. 5 D), structures which develop from a *dlx5a*-positive domains (Fig. S3) [38], [49].

The present data demonstrate that *dlx5a/6a* genes are required, directly or indirectly, for osteoblast differentiation of the cleithrum in zebrafish.

### Knock down of *dlx5a/6a* impacts on median fin fold morphogenesis

**Figure 6:**
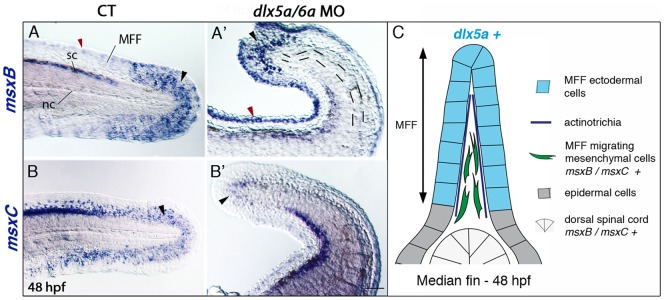
Impaired median fin fold expression of *msx* genes in *dlx5a/6a* morphants. Whole mount *in situ* hybridization for *msxB* (A, A′) *and msxC* (B, B′) in lateral views of the posterior axis of control (A–B) and *dlx5a/6a* morphant (A′–B′) embryos at 48 hpf. In controls, *msxB* and *msxC* genes are expressed in the spinal cord (sc) and in mesenchymal cells of the median fin fold (MFF) (black arrowheads A–B). Slight *msxB* expression is also observed in MFF apical cells (red arrowhead A). In morphants, *msx* expression is limited to a few distal MFF mesenchymal cells (black arrowheads A′–B′) and aberrant *msxB* expression is detected in the MFF apical cells (red arrowhead A′). The dashed lines in (A′) underlie the undulating and larger notochord (nc) displayed in the morphants. (C) Schematic representation of the dorsal median fin at 48 hpf summarizing the expression of *dlx5a* and *msx* genes in their corresponding cellular types. Scale bar shown in B′ for all panels 50 µm.

To study the effects of the *dlx5a/6a* knock down in unpaired fin development, we analyzed the expression of *bmp4*, *fgf8a*, *and1*, *msxB* and *msxC* genes in the median fin fold (MFF) of *dlx5a/6a* morphants at 16 hpf, 24 hpf (during MFF specification) and 48 hpf (during MFF morphogenesis). At 16 hpf and 24 hpf, no notable changes in *bmp4*, *fgf8a* and *msx* expression in the median fin fold are observed in the morphants compared to controls (Table 1, data not shown). At 48 hpf, *msxB* is expressed in distal mesenchymal cells of the MFF whereas *msxC* shows a larger anterior expression domain (Fig. 6 A–B, black arrowheads). Both transcripts are also detected in the spinal cord. In *dlx5a/6a* morphant embryos, *msxB* and *msxC* expression is still present in the spinal cord, but only a few *msx*-positive cells are detected in mesenchymal cells in the distal part of the MFF (Fig. 6 A′–B′, black arrowheads), whereas aberrant *msxB* expression is detected in MFF apical ectodermal cells (Fig. 6 A′, red arrowhead). In contrast, the *fgf8a* expression initially observed in MFF ectodermal cells of controls at 48 hpf is not affected in the morphants (Table 1, data not shown). The latter results suggested developmental defects of MFF mesenchymal cells.

**Table 1:** Markers affected by the *dlx5a/6a* knock down during the early development of pectoral and median fins.

	Pectoral fin		Median fin		
	24 hpf	48 hpf	16 hpf	24 hpf	48 hpf
***bmp4***	A	A	U	U	X
***fgf8a***	X	A	X	U	U
***msxB***	A	A	U	U	A
***msxC***	A	A	U	U	A
***and1***	X	A	X	A	A

A, affected; U, unaffected; X, not expressed.

**Figure 7:**
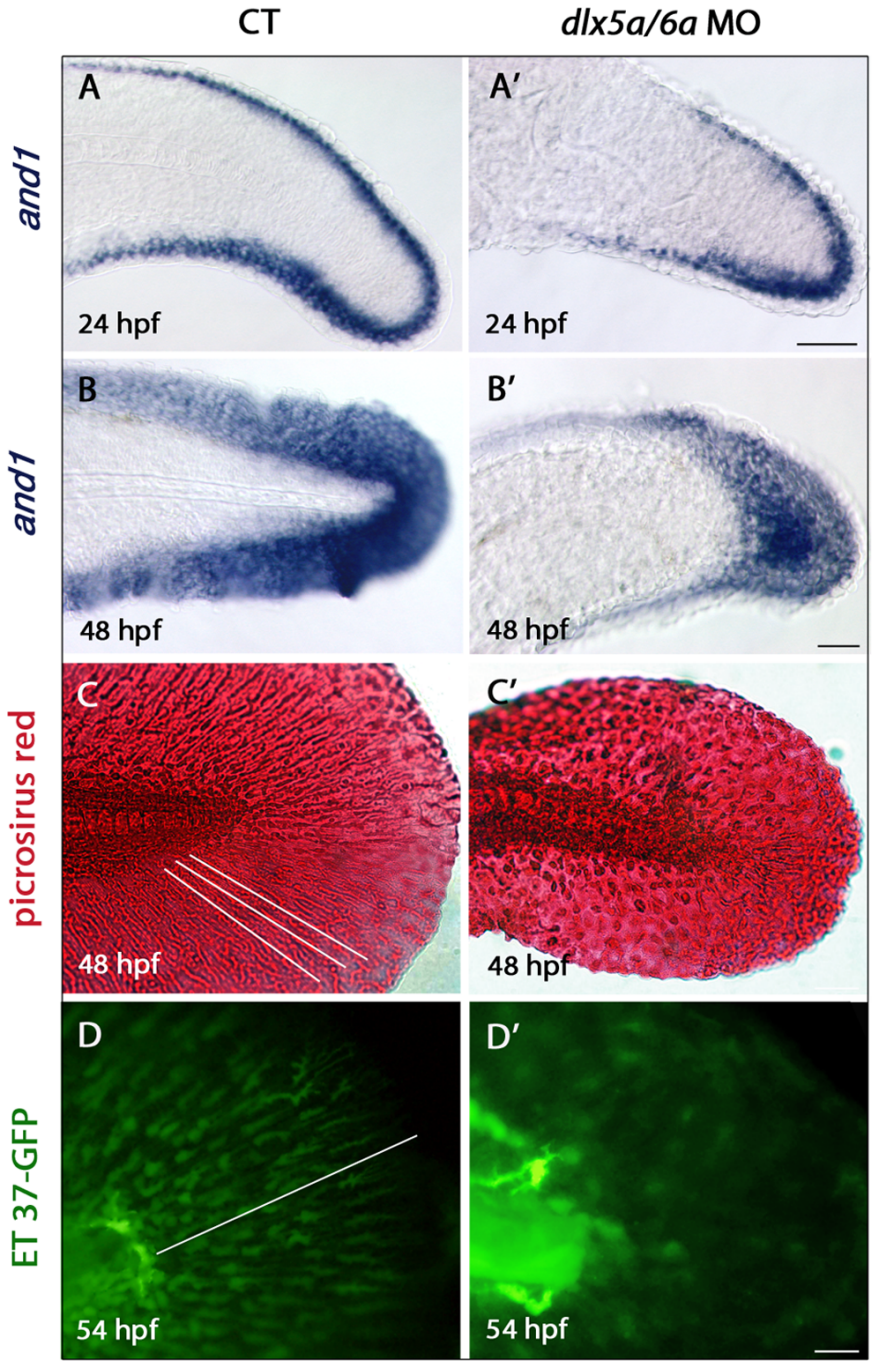
Defects in actinotrichia formation in the median fin fold of *dlx5a/6a* morphants. Lateral views of the developing median fin fold (MFF) of control (A–D) and *dlx5a/6a* morphant (A′–D′) embryos. Whole mount *in situ* hybridization for *and1* at 24 (A, A′) and 48 hpf (B, B′). In control embryos, *and1* is expressed in epithelial cells of the fin fold at 24 hpf and in all MFF cells at 48 hpf (A–B). In *dlx5a/6a* morphants, *and1* expression is detected in the MFF but transcripts show a less extended antero-posterior expression domain at 24 hpf (A′). At 48 hpf, morphants display a thinner *and1* expression domain in the ventral and dorsal part of the MFF (B′). The picrosirius red histological staining in 48 hpf control embryos reveals the MFF cellular organisation with the presence of actinotrichia (C, the white lines indicate their orientation and length). In the morphants, the MFF is smaller and granular associated with absence of actinotrichia (C′). The ET 37-GFP enhancer-trap line reveals the MFF mesenchymal cells displaying filopodia which migrate along the actinotrichia at 54 hpf (D, the white line indicates the direction of migration). The *dlx5a/6a* knock down in ET 37-GFP embryos leads to impaired MFF mesenchymal migration, the cells are disorganized and do not show filopodia. Scale bars in A–B 50 µm, C 20 µm, D 10 µm.

The MFF of *dlx* morphants presents a granular aspect which seems to be associated with absence of actinotrichia. The actinotrichia are non-calcified fibrils which develop in the early zebrafish fins. They act as a scaffold for the migration of mesenchymal cells and, later, for fin ray formation during zebrafish fin skeletogenesis. The actinotrichia defects in our morphants were further investigated by examining *and1* expression during posterior axis development. The *and1* gene is a member of the actinodin family which encodes proteins that are essential structural components of zebrafish fin rays and fin folds and required for actinotrichia formation [46]. In control embryos, *and1* expression is observed in epithelial cells of the fin fold at 24 hpf and in all MFF cells at 48 hpf (Fig. 7 A–B). As previously reported [46], *and1* transcripts are not co-expressed with *dlx5a* in the apical ectodermal cells at 24 hpf, but rather in epithelial cells directly adjacent to the apical ectodermal tissue (Fig. S4 A). In 24 hpf *dlx5a/6a* morphants, *and1* is detected in the MFF but transcripts show a shorter antero-posterior expression domain (Fig. 7 A′, Fig. S4 B). At 48 hpf, transcripts are also observed in a less extended MFF domain (Fig. 7 B′).

We then performed picrosirius red staining to analyze the cellular organization of the median fin fold and to reveal the presence of actinotrichia. In CT embryos at 48 hpf, actinotrichia are clearly visible (Fig. 7 C, the white lines indicate their orientations and lengths). In *dlx5a/6a* morphants, actinotrichia do not form and the fin fold is smaller and granular.

We used the zebrafish ET-37 enhancer-trap line [55], expressing GFP in migrating mesenchymal cells of the fin fold, to examine MFF cell migration following *dlx5a/6a* knock down. In 54 hpf control embryos, migrating mesenchymal cells in the MFF are aligned and show filopodia orientated in the direction of migration (Fig. 7 D, the white line indicates the direction of migration). In *dlx5a/6a* morphants, mesenchymal cells are disorganized and do not show filopodia (Fig. 7 D–D′). Moreover, the median fin fold phenotype observed in the double morphants is associated with increased cell death and decreased proliferation at 24 hpf (Fig. S5).

### *dlx5a* is expressed during fin skeleton formation

Several studies in tetrapod species have demonstrated the role of *Dlx5/6* genes in endochondral bone development [14], [56], [57]. The expression of *dlx5a/6a* has also been reported in some craniofacial bones that develop by either endochondral or intramembranous ossification [38], [49]. To further examine the potential implication of *dlx5a* in appendage skeletogenesis, we completed *dlx5a* expression analysis at later stages during fin bone formation. We performed *in situ* hybridization on sections of wild-type larvae from around 6 days (4.2 mm) to one month post fertilization (8.7 mm).

**Figure 8:**
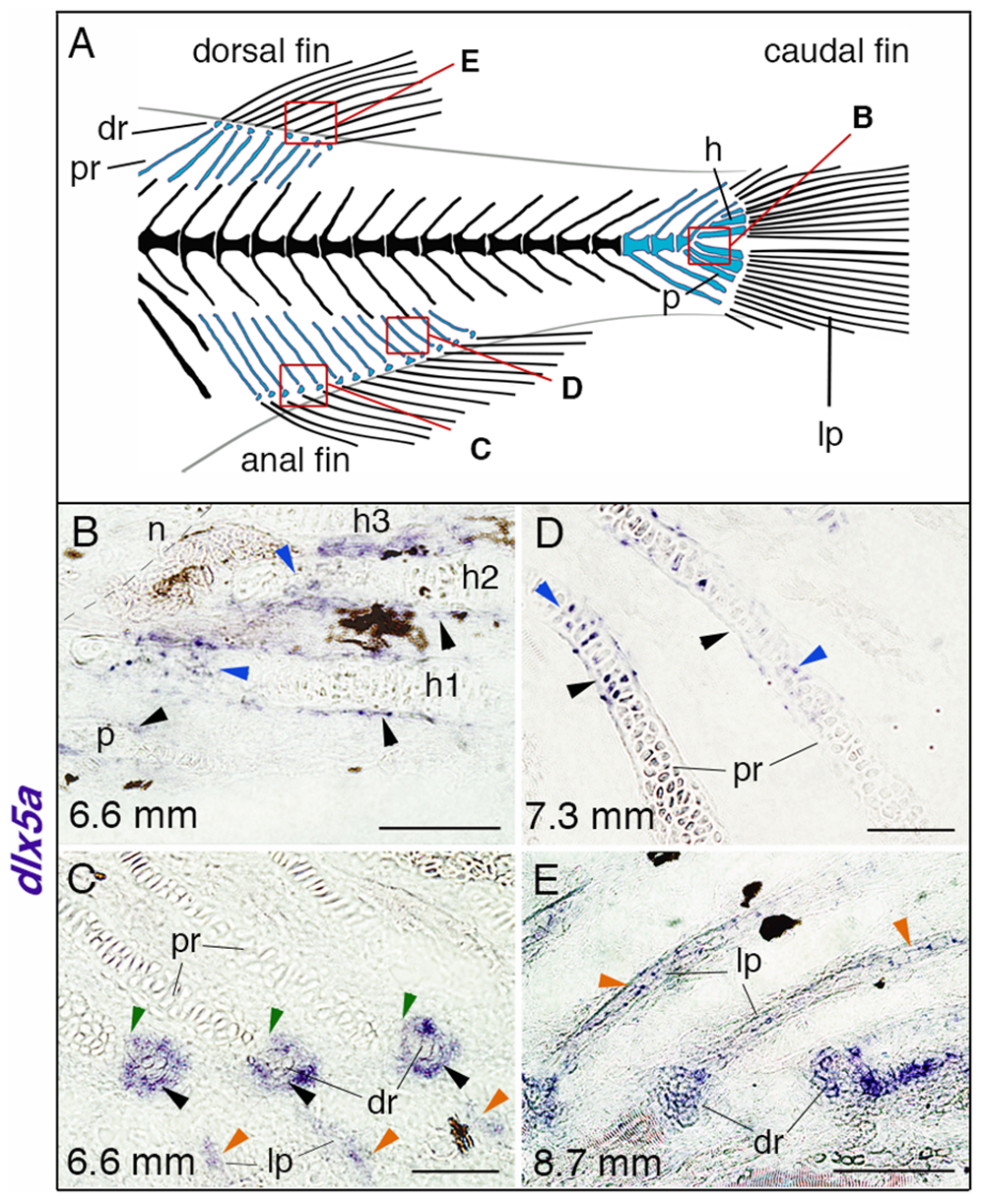
*dlx5a* expression during unpaired fin skeletogenesis. (A) Overview of the posterior axial skeleton of a one-month-old zebrafish. Endoskeletal fin supports are colored in blue and red squares indicate the structures analyzed in (B–E). (B–E) Whole mount *in situ* hybridization for *dlx5a* on 10 µm parasagittal frozen sections of late-stage zebrafish from 6.6 mm (16 days post-fertilization) to 8.7 mm (1 month post-fertilization). In 6.6 mm larvae, *dlx5a* is expressed in the perichondrium of the hypurals 1 to 3 (h1-3) and of the parahypural (p) (B, black arrowheads) as well as in maturing chondrocytes of h1 and h2 (B, blue arrowheads). The transcripts are also detected in cells surrounding the distal radials (dr) during radial segmentation (C, black arrowheads), notably in the zone of segmentation (C, green arrowheads), and in the developing lepidotrichia (lp, C, orange arrowheads). Later, *dlx5a* expression is observed in the perichondrium (D, black arrowheads) and in maturing chondrocytes (blue arrowheads) of proximal radials (pr) of 7.3 mm larvae. In 8.7 mm larvae, *dlx5a* expression is maintained in the well-developed lepidotrichia (lp) (E, orange arrowheads). Black-brown patches in B–C, E are melanophores. Scale bars B, E 20 µm, C–D 10 µm.

Expression of *dlx5a* was first detected in the perichondrium of the hypurals in 6.2 mm larvae (data not shown) whereas no expression was observed in the forming radials. Slightly later, in 6.6 mm larvae, *dlx5a* expression is maintained in the parahypural and hypural perichondrium (Fig. 8 B, black arrowheads) and transcripts are also observed in maturing chondrocytes (Fig. 8 B, blue arrowheads). In the same specimen, *dlx5a* is expressed in cells surrounding the distal radials during radial segmentation (Fig. 8 C, black arrowheads), including in the zone of segmentation (ZS) (Fig. 8 C, green arrowheads), and in developing lepidotrichia of the anal fin (Fig. 8 C, orange arrowheads), but not in the proximal radials (Fig. 8 C). Expression of *dlx5a* in the ZS is still detected in 7.3 mm larvae while radial segmentation is almost completed (Fig. S6 B, blue arrowheads) but not in 8.7 mm larvae after radial segmentation (Fig. S6 C, black asterisks), whereas expression is maintained but decreased in cells surrounding the distal radials (Fig. S6 C). Moreover, *dlx5a* transcripts are observed in maturing chondrocytes (Fig. 8 D, blue arrowheads) and in the flanking perichondrium of anal proximal radials in 7.3 mm larvae (Fig. 8 D, black arrowheads). Later, *dlx5a* expression is detected in well-developed lepidotrichia of the dorsal fin in 8.7 mm fish (Fig. 8 E, orange arrowheads). The results suggest that *dlx5a* is implicated in the formation of skeletal components of the developing zebrafish fins.

## Discussion

### *dlx5a/6a* genes are essential for the initiation of pectoral fin development

During zebrafish development, the three *dlx* bigene clusters, *dlx1a/2a*, *dlx3b/4b* and *dlx5a/6a*, are expressed in fin primordia [16]. However, no fin phenotypes have been observed in *dlx1a/2a and dlx3b/4b* morphants (E. Heude unpublished observations, personal communication from A. Fritz, [30], [58]). In mouse, combinatorial mutations of several *Dlx* genes have shown that only *Dlx5/6* and *Dlx2/5* mutants display distal limb malformations [14], [36], [59]. Thus, the *Dlx5/Dlx6* genes seem to play a critical role in the formation of appendages in vertebrates.

In zebrafish, the *dlx5a/6a* genes are early markers of apical ectodermal cells of the presumptive and developing pectoral fin buds and median fin fold (Figs. 1, 2, Figs. S1, S2) suggesting their role in early specification and morphogenesis of these structures.

At the pectoral level, *dlx5a* is expressed during pectoral fin bud initiation at 24 hpf and the *dlx5a/6a* knock down is associated with agenesis of pectoral fin buds (Figs. 3, 4). The data indicate that *dlx5a/6a* genes are required for pectoral fin bud induction and outgrowth in zebrafish. We show that *dlx5a* expression in the pectoral fin fold progressively decreases from 36 hpf to 48 hpf to become hardly detectable at 72 hpf (Fig. 2). Interestingly, this period corresponds to the AER/PFF transition followed by PFF elongation [20]. It has been shown that the unilateral surgical ablation of the PFF induces an activation of *dlx5a* expression in the reformed AER, whereas *dlx5a* is no longer detected in the control PFF in zebrafish [20]. The data suggest that *dlx5a* is essential for the first step of pectoral fin specification and morphogenesis until AER/PFF transition. In mouse, the *Dlx5/6* genes are expressed in the AER of limb buds from E9.5 to E12.5 [13]–[15]. However, the genes are not expressed in the limb field during limb bud induction at E8.5 (E. Heude unpublished observations, [60]). The data indicate that *Dlx5/6* are not involved in the early specification of limb buds in mouse. Indeed, in contrast to what is observed in *dlx5a/6a* morphants, the *Dlx5/6* null mutant mice do not show limb bud agenesis, the *Dlx5/6* inactivation only alters limb morphogenesis.

The *dlx5a/6a* knock down leads to severe decreases or loss of expression of *bmp4*, *fgf8a*, *and1* and *msx* genes. The *Dlx5/6* null mutant mice show a loss of *Bmp4*, *Fgf8* and *Msx2* expression in the medial part of the limb buds [14], [15]. A recent study demonstrated that the Dlx5 protein binds to conserved sequences in the proximity of the *Bmp2* and *Bmp4* loci in vitro, suggesting a direct regulation of *Bmp* genes [15]. Moreover, BMPs appear to act as signaling relays between *Dlx* and *Msx* genes during ectoderm-mesoderm communication for proper limb development in the mouse [15]. *Dlx5* seems to have a key role in the initiation of an ectoderm-mesoderm dialog during tetrapod limb morphogenesis. Our results suggest that *dlx5a/6a* expression in ectodermal cells acts upstream of *bmp4, fgf8a* and *msx* genes in the pectoral fin bud primordia to induce pectoral fin outgrowth in zebrafish. The *dlx5a/6a* genes seem to be crucial to induce the ectoderm-mesoderm communication at the basis of appendage outgrowth in zebrafish. However, it appears that the role of *Dlx5/6* in the early development of paired appendages diverged between teleosts and tetrapods during vertebrate evolution.

### Distinct requirement for *dlx5a/6a* gene function during paired and unpaired fin development

It has been shown that the early dorsal MFF apical tissue consists of epidermal cells covered by periderm [17], [61]. Our results show that, at the posterior level, *dlx5a* is first detected in ectodermal cells underlying the periderm at the lateral edges of the neural keel, with expressing cells later converging towards the midline to cover the posterior neural rod (Fig. 1) [62], [63]. From 15.5 hpf to 16 hpf, we show that ectodermal cells expressing *dlx5a* are laterally connected to the neural ectoderm. Thus, we propose that the presumptive dorsal MFF territory expressing *dlx5a* results from the medial fusion of the lateral edges of the neural keel during neural rod formation (Fig. 1 D).

We show that the *dlx5a/6a* knock down leads to impaired median fin fold development associated with *msx* and *and1* misexpression in MFF mesenchymal cells, increased apoptosis and decreased proliferation in the MFF at 24 hpf (Figs. 6, 7 A, A′–B, B′, Figs. S4, S5). Moreover, similar to what was observed in *and1/and2* double morphants [46], the median fin fold anomalies of *dlx5a/6a* morphants are characterized by defective mesenchymal migration and absence of actinotrichia (Fig. 7 C, C′–D, D′). As already mentioned, *and1* is expressed in cells adjacent to the *dlx5a*-expressing apical ectodermal tissue in the MFF at 24 hpf. Our results suggest that the *dlx5a/6a* genes may be required to maintain proper *and1* expression leading to actinotrichia formation during early MFF development.

The pectoral fin bud and median fin fold share similar embryonic components including apical ectodermal tissue and mesenchymal cells migrating along the actinotrichia to invade the folds (Fig. 4 I, Fig 6. C). Both structures express a similar set of genes in the corresponding embryonic tissues. Indeed, early *dlx5a* expression is detected in apical ectodermal cells of the presumptive MFF at 15.5 hpf and in the PFF at 24 hpf suggesting a similar role for *dlx5a* in the specification of both structures. However, whereas the *dlx5a/6a* knock down leads to the early loss of expression of markers associated with fin bud agenesis at the pectoral level, we did not observe obvious differences in the expression of *bmp4*, *fgf8a* and *msx* genes in the MFF of the double morphants compared to controls at 16 hpf and 24 hpf (Table 1). Moreover, the median fin fold begins to develop in *dlx5a/6a* morphants and later shows developmental defects at 48 hpf. In contrast to what we described at the pectoral level, *dlx5a/6a* genes seem to be required for proper morphogenesis of the MFF but not for its specification.

In pectoral and median fin buds, *dlx5a* expression is associated, directly or indirectly, with activation of *bmp4*, *fgf8a*, *msx* and *and1* genes. Fgf8 and Dlx proteins are also detected in the MFF apical tissue of sharks suggesting that ancestral molecular mechanisms implicated in unpaired fin development might have been established during early vertebrate evolution [22]. It is now well accepted that paired appendages evolved after unpaired appendages [22], [64], [65]. Despite their different embryonic origins [66], [67], it has been suggested that paired and unpaired fins use a common suite of developmental mechanisms, a hypothesis mainly based on expression analyses [17], [22], [68], [69]. The latter studies support that ancestral mechanisms of median fin development have been co-opted for the development of paired appendages. In contrast, our observations show that *dlx5a/6a* knock down has a different impact on zebrafish pectoral and median fin development (Table 1). The early communication between MFF apical ectodermal cells and underlying structures seems to take place in *dlx5a/6a* morphants. However, *dlx5a/6a* expression in apical ectodermal cells may be required for the specification of the pectoral fin bud. Therefore, although development of paired and unpaired fins may use similar molecular mechanisms, when it comes to those involving *dlx5a/6a*, differences in timing, expression territory or usage of target genes may underlie the profound phenotypic differences that we observed. Alternatively, paired and unpaired fins may use different *dlx5a/6a*-associated molecular mechanism for their development. The differential effects of zebrafish mutations affecting other genes on pectoral and median fin development also support differences in mechanisms (for review, [70]).

### Cleithrum formation requires *dlx5a/6a* expression

We show that *dlx5a* is highly expressed in the differentiating cleithrum from 36 hpf to 72 hpf in control embryos (Fig. 2 C–D). The cleithrum extends from the base of the pectoral fin and forms the posterior edge of the gill chamber. The cleithrum is an ancestral component of the pectoral girdle not homologous to any bones in mammals. It is present in all bony fish ancestors (Osteichthyes) and stem-group tetrapods but is absent in living tetrapods [71], except frogs [72]. Apparently, loss of the cleithrum during vertebrate evolution corresponds to the appearance of neck structures for head mobility. In zebrafish, the cleithrum is a dermal bone from mesodermal origin [1], [73] and is the first bone to mineralize at the axial level during early zebrafish development. Expression of *dlx5a* in the cleithrum is associated with expression of *runx2b* at 36 hpf and *runx2b and col10a1* at 48 hpf, early/intermediate and intermediate/late markers of osteoblast differentiation respectively (Fig. 5 A, C, E) [47], [53], [54]. The expression analysis indicates that *dlx5a* is an early/intermediate marker of differentiating cleithrum osteoblasts.

In *dlx5a*/*6a* morphants, expression of *runx2b* and *col10a1* is lost suggesting impaired cleithrum development (Fig. 5 B, D, F). Absence of cleithrum is not an indirect effect of the loss of pectoral fins in *dlx5a/6a* morphants. Many studies reported experiments that led to the absence of pectoral fins while the cleithrum was still present, often without any signs of dysmorphologies [10], [11], [74]–[77]. Moreover, *dlx5a/6a* knock down also leads to a loss of *runx2b* expression in the opercular and ceratobranchial bones (Fig. 5 C, D), skeletal structures which both develop in a *dlx5a/6a*-positive context at the craniofacial level (Fig. S3) [38], [49]. Our results show that *dlx5a/dlx6a* genes are required for cleithrum formation in zebrafish.

### *dlx5a* is involved in fin skeletogenesis

To further study the potential implication of *dlx5a* during zebrafish fin skeletogenesis, we extended *dlx5a* expression analysis to later stages of unpaired fin bone formation. The fin skeleton is a mix of endochondral and dermal bones; the radial and hypural bones which support and articulate the fins rays originate from cartilaginous structures (Fig. 8 A, blue structures), whereas the lepidotrichia (fin rays) are dermal bones [65]. We show that *dlx5a* is expressed in the developing endochondral and dermal structures (Fig. 8, Fig. S6). During endochondral fin skeletogenesis, *dlx5a* is expressed in maturing chondrocytes and in the perichondrium of hypurals and proximal radials (Fig. 8 B, D). We never detected transcripts in proliferating or resting chondrocytes. The results are consistent with what is observed during long bone formation in tetrapods and suggest a conserved *Dlx5* implication in chondrocyte differentiation among vertebrate species.

In teleosts, the radials arise from a common mesenchymal condensation which later segments into proximal and distal components. It has been suggested that fish radial segmentation corresponds to tetrapod joint interzone formation and that both processes share similar spatio-temporal expression of genes between zebrafish, chick and mouse [78]. Our results show that *dlx5a* expression is detected in cells surrounding the distal radials, notably in the zone of segmentation (ZS) during radial segmentation suggesting its role in the latter process (Fig. 8 C, Fig. S6 B). It has been shown that *Dlx5/6* genes are early markers of the presumptive elbow joint in chick developing limbs [79]. The study reveals that co-expression of *Dlx5* and *Gdf5*, a gene known to regulate joint formation, corresponds to the initiation of elbow joint formation. Interestingly, *gdf5* is also observed in the ZS in 6.6 mm zebrafish larvae [80] at the same stage as *dlx5a* transcripts (Fig. 8 C). Our data further support the potential implication of *Dlx5* in appendage segmentation/joint formation in vertebrates. In parallel, we observed that *dlx5a* is expressed in the developing lepidotrichia of median fins (Fig. 8 C, E). Altogether, the results associated with what is observed in the cleithrum (Fig. 5) indicate that *dlx5a* is involved in zebrafish osteoblast differentiation. However, the role of *dlx5a* in zebrafish skeletogenesis requires further investigations.

## Conclusion

Our results demonstrate that *dlx5a/6a* genes are necessary for the specification and outgrowth of the zebrafish pectoral fin buds. However, the *dlx5a/6a* genes do not seem to carry out the same role during the development of pectoral and median fins suggesting differences in the molecular mechanisms controlling the early development of paired and unpaired fins. The origin of paired fins during vertebrate evolution is still controversial [22], [66], [67], [69]. Our results can refocus arguments and may open new evolutionary perspectives on the mechanistic basis of paired appendage genesis in vertebrate species.

## Supporting Information

Figure S1 **Expression patterns of** ***dlx5a*** **during zebrafish median fin development.** Whole mount *in situ* hybridization for *dlx5a* on lateral view of the posterior axis of 24 hpf (A) and 48 hpf (B) zebrafish embryos and on 10 µm parasagittal frozen sections (A′, B′) at the level indicated in (A, B). At 24 and 48 hpf, *dlx5a* expression is limited to apical ectodermal cells of the median fin fold (MFF). nc, notochord; nt, neural tube; sc, spinal cord. Scale bars 50 µm.
(PDF)

Figure S2 **Comparison of** ***dlx5a*** **and** ***dlx6a*** **expression in the developing zebrafish fins.** Whole mount *in situ* hybridization for *dlx5a* (A, B) and *dlx6a* (C, D) in the pectoral fin bud (PFB) (A, C) and in the median fin fold (MFF) (B, D) of 30 hpf embryos. Expression of *dlx5a* is detected in apical ectodermal cells of both pectoral and median developing fins (A, B, black arrowheads). Expression of *dlx6a* mirrors *dlx5a* expression, however *dlx6a* transcripts seem to be present at lower level (C, D, black arrowheads). Scale bars 50 µm.
(PDF)

Figure S3 **The opercle, the ceratobranchial-5 bone and the cleithrum all develop in a** ***dlx5a*** **-positive context.** Dorsal and lateral views of whole mount *in situ* hybridization for *runx2b* (A, A′) and *dlx5a* (B, B′) in the anterior region of 48 hpf zebrafish embryos. Expression of *runx2b* reveals the opercle (op), the ceratobranchial-5 bone (cb5) and the cleithrum (cl) (A, A′), structures which differentiate at early stage of zebrafish development. The *dlx5a* expression analysis at equivalent stage shows that the three bones develop in *dlx5a*-positive domains, explaining the loss of *runx2b* expression in *dlx5a/6a* morphants at the pectoral and craniofacial levels shown in Fig. 5 D (black and blue asterisks). ba, branchial arches. Scale bars 100 µm.
(PDF)

Figure S4 **Expression of** ***and1*** **in the median fin fold of control and** ***dlx5a/6a*** **morphant embryos.** Ventral view at the posterior level of whole mount *in situ* hybridization for *and1* in 24 hpf controls (A) and *dlx5a/6a* morphants (B). The ventral view reveals that *and1* transcripts are not expressed in the apical ectodermal cells of the median fin fold (medial line, AP), but in ectodermal cells adjacent to the AP. Scale bar shown in B for the two panels 50 µm.
(PDF)

Figure S5 **The median fin fold defects in** ***dlx5a/6a*** **morphants are associated with altered cell proliferation and apoptosis.** Lateral view at the posterior axis of BrdU (A, A′) and TUNEL (B, B′) assays on control (A–B) and *dlx5a/6a* morphant (A′–B′) embryos at 24 hpf. The BrdU assay shows that controls present proliferating cells in the median fin fold whereas no BrdU-positive cells are observed in the morphants. In parallel, the morphants show a high increase of apoptotic cells in the MFF compared to controls (B–B′). Scale bar shown in B′ for all panels 20 µm.
(PDF)

Figure S6 **Expression of** ***dlx5a*** **in fin skeletal components of late-stage zebrafish larvae.** (A) Overview of the posterior axial skeleton of a one-month-old zebrafish. Endoskeletal fin supports are colored blue and red square indicates the structures analyzed in (B–C). (B, C) Whole mount *in situ* hybridization for *dlx5a* on 10 µm parasagittal frozen sections of 7.3 mm and 8.7 mm late-stage zebrafish. As seen in Fig. 8 C in 6.6 mm larvae, *dlx5a* expression is still detected in cells surrounding the distal radials (dr) in 7.3 mm larvae, including in the zone of segmentation (ZS) (B, blue arrowheads) when the radial segmentation is almost completed. Later, after segmentation (8.7 mm), *dlx5a* expression is maintained but decreases in cells surrounding the distal radials and is no longer detected in the ZS (black asterisks) (C). dr, distal radials; lp, lepidotrichia; pr, proximal radials. Scale bars B–C 10 µm.
(PDF)

Checklist S1 **ARRIVE Checklist.**
(DOC)

## References

[1] Grandel H, Schulte-Merker S (1998) The development of the paired fins in the zebrafish (*Danio rerio*). Mech Dev 79: 99–120. 10349624 10.1016/s0925-4773(98)00176-2

[2] Zakany J, Kmita M, Duboule D (2004) A dual role for *Hox* genes in limb anterior-posterior asymmetry. Science 304: 1669–1672. 15192229 10.1126/science.1096049

[3] Freitas R, Zhang G, Cohn MJ (2007) Biphasic *Hoxd* gene expression in shark paired fins reveals an ancient origin of the distal limb domain. PLoS One 2: e754. 17710153 10.1371/journal.pone.0000754 PMC1937022

[4] Capdevila J, Izpisua Belmonte JC (2001) Patterning mechanisms controlling vertebrate limb development. Annu Rev Cell Dev Biol 17: 87–132. 11687485 10.1146/annurev.cellbio.17.1.87

[5] Ahn D, Ho RK (2008) Tri-phasic expression of posterior *Hox* genes during development of pectoral fins in zebrafish: implications for the evolution of vertebrate paired appendages. Dev Biol 322: 220–233. 18638469 10.1016/j.ydbio.2008.06.032

[6] Riddle RD, Johnson RL, Laufer E, Tabin C (1993) *Sonic hedgehog* mediates the polarizing activity of the ZPA. Cell 75: 1401–1416. 8269518 10.1016/0092-8674(93)90626-2

[7] Grandel H, Draper BW, Schulte-Merker S (2000) *dackel* acts in the ectoderm of the zebrafish pectoral fin bud to maintain AER signaling. Development 127: 4169–4178. 10976049 10.1242/dev.127.19.4169

[8] Ruvinsky I, Gibson-Brown JJ (2000) Genetic and developmental bases of serial homology in vertebrate limb evolution. Development 127: 5233–5244. 11076746 10.1242/dev.127.24.5233

[9] Garrity DM, Childs S, Fishman MC (2002) The heartstrings mutation in zebrafish causes heart/fin *Tbx5* deficiency syndrome. Development 129: 4635–4645. 12223419 10.1242/dev.129.19.4635

[10] Fischer S, Draper BW, Neumann CJ (2003) The zebrafish *fgf24* mutant identifies an additional level of Fgf signaling involved in vertebrate forelimb initiation. Development 130: 3515–3524. 12810598 10.1242/dev.00537

[11] Norton WH, Ledin J, Grandel H, Neumann CJ (2005) HSPG synthesis by zebrafish Ext2 and Extl3 is required for Fgf10 signalling during limb development. Development 132: 4963–4973. 16221725 10.1242/dev.02084

[12] Simeone A, Acampora D, Pannese M, D'Esposito M, Stornaiuolo A, et al (1994) Cloning and characterization of two members of the vertebrate *Dlx* gene family. Proc Natl Acad Sci U S A 91: 2250–2254. 7907794 10.1073/pnas.91.6.2250 PMC43348

[13] Acampora D, Merlo GR, Paleari L, Zerega B, Postiglione MP, et al (1999) Craniofacial, vestibular and bone defects in mice lacking the *Distal-less-related* gene *Dlx5* . Development 126: 3795–3809. 10433909 10.1242/dev.126.17.3795

[14] Robledo RF, Rajan L, Li X, Lufkin T (2002) The *Dlx5* and *Dlx6* homeobox genes are essential for craniofacial, axial, and appendicular skeletal development. Genes Dev 16: 1089–1101. 12000792 10.1101/gad.988402 PMC186247

[15] Vieux-Rochas M, Bouhali K, Mantero S, Garaffo G, Provero P, et al (2013) BMP-mediated functional cooperation between Dlx5;Dlx6 and Msx1;Msx2 during mammalian limb development. PLoS One 8: e51700. 23382810 10.1371/journal.pone.0051700 PMC3558506

[16] Ellies DL, Stock DW, Hatch G, Giroux G, Weiss KM, et al (1997) Relationship between the genomic organization and the overlapping embryonic expression patterns of the zebrafish *dlx* genes. Genomics 45: 580–590. 9367683 10.1006/geno.1997.4978

[17] Abe G, Ide H, Tamura K (2007) Function of FGF signaling in the developmental process of the median fin fold in zebrafish. Dev Biol 304: 355–366. 17258191 10.1016/j.ydbio.2006.12.040

[18] Kouwenhoven EN, van Heeringen SJ, Tena JJ, Oti M, Dutilh BE, et al (2010) Genome-wide profiling of p63 DNA-binding sites identifies an element that regulates gene expression during limb development in the 7q21 SHFM1 locus. PLoS Genet 6: e1001065. 20808887 10.1371/journal.pgen.1001065 PMC2924305

[19] Birnbaum RY, Everman DB, Murphy KK, Gurrieri F, Schwartz CE, et al (2012) Functional characterization of tissue-specific enhancers in the *DLX5/6* locus. Hum Mol Genet 21: 4930–4938. 22914741 10.1093/hmg/dds336 PMC3529576

[20] Yano T, Abe G, Yokoyama H, Kawakami K, Tamura K (2012) Mechanism of pectoral fin outgrowth in zebrafish development. Development 139: 2916–2925. 22791899 10.1242/dev.075572

[21] Zeller R, Lopez-Rios J, Zuniga A (2009) Vertebrate limb bud development: moving towards integrative analysis of organogenesis. Nat Rev Genet 10: 845–858. 19920852 10.1038/nrg2681

[22] Freitas R, Zhang G, Cohn MJ (2006) Evidence that mechanisms of fin development evolved in the midline of early vertebrates. Nature 442: 1033–1037. 16878142 10.1038/nature04984

[23] Cohen S, Bronner G, Kuttner F, Jurgens G, Jackle H (1989) *Distal-less* encodes a homeodomaine protein required for limb development in *Drosophila* . Nature 338: 432–434. 2564639 10.1038/338432a0

[24] Stock DW, Ellies DL, Zhao Z, Ekker M, Ruddle FH, et al (1996) The evolution of the vertebrate *Dlx* gene family. Proc Natl Acad Sci U S A 93: 10858–10863. 8855272 10.1073/pnas.93.20.10858 PMC38247

[25] Zerucha T, Ekker M (2000) *Distal-less-related* homeobox genes of vertebrates: evolution, function, and regulation. Biochem Cell Biol 78: 593–601. 11103950

[26] Quint E, Zerucha T, Ekker M (2000) Differential expression of orthologous *Dlx* genes in zebrafish and mice: implications for the evolution of the *Dlx* homeobox gene family. J Exp Zool 288: 235–241. 11069141 10.1002/1097-010x(20001015)288:3<235::aid-jez4>3.0.co;2-j

[27] Debiais-Thibaud M, Germon I, Laurenti P, Casane D, Borday-Birraux V (2008) Low divergence in *Dlx* gene expression between dentitions of the medaka (*Oryzias latipes*) versus high level of expression shuffling in osteichtyans. Evol Dev 10: 464–476. 18638323 10.1111/j.1525-142X.2008.00257.x

[28] MacDonald RB, Debiais-Thibaud M, Martin K, Poitras L, Tay BH, et al (2010) Functional conservation of a forebrain enhancer from the elephant shark (*Callorhinchus milii*) in zebrafish and mice. BMC Evol Biol 10: 157. 20504318 10.1186/1471-2148-10-157 PMC2891724

[29] MacDonald RB, Debiais-Thibaud M, Talbot JC, Ekker M (2010) The relationship between *dlx* and *gad1* expression indicates highly conserved genetic pathways in the zebrafish forebrain. Dev Dyn 239: 2298–2306. 20658694 10.1002/dvdy.22365 PMC3097087

[30] Macdonald RB, Pollack JN, Debiais-Thibaud M, Heude E, Coffin Talbot J, et al (2013) The *ascl1a* and *dlx* genes have a regulatory role in the development of GABAergic interneurons in the zebrafish diencephalon. Dev Biol 381: 276–285. 23747543 10.1016/j.ydbio.2013.05.025 PMC3750962

[31] Depew MJ, Lufkin T, Rubenstein JL (2002) Specification of jaw subdivisions by *Dlx* genes. Science 298: 381–385. 12193642 10.1126/science.1075703

[32] Beverdam A, Merlo GR, Paleari L, Mantero S, Genova F, et al (2002) Jaw transformation with gain of symmetry after *Dlx5/Dlx6* inactivation: mirror of the past? Genesis 34: 221–227. 12434331 10.1002/gene.10156

[33] Heude E, Bouhali K, Kurihara Y, Kurihara H, Couly G, et al (2010) Jaw muscularization requires *Dlx* expression by cranial neural crest cells. Proc Natl Acad Sci U S A 107: 11441–11446. 20534536 10.1073/pnas.1001582107 PMC2895105

[34] Merlo GR, Paleari L, Mantero S, Genova F, Beverdam A, et al (2002) Mouse model of split hand/foot malformation type I. Genesis 33: 97–101. 12112878 10.1002/gene.10098

[35] Kraus P, Lufkin T (2006) *Dlx* homeobox gene control of mammalian limb and craniofacial development. Am J Med Genet A 140: 1366–1374. 16688724 10.1002/ajmg.a.31252

[36] Panganiban G, Rubenstein JL (2002) Developmental functions of the *Distal-less/Dlx* homeobox genes. Development 129: 4371–4386. 12223397 10.1242/dev.129.19.4371

[37] Lo Iacono N, Mantero S, Chiarelli A, Garcia E, Mills AA, et al (2008) Regulation of Dlx5 and Dlx6 gene expression by p63 is involved in EEC and SHFM congenital limb defects. Development 135: 1377–1388. 18326838 10.1242/dev.011759

[38] Talbot JC, Johnson SL, Kimmel CB (2010) *hand2* and *Dlx* genes specify dorsal, intermediate and ventral domains within zebrafish pharyngeal arches. Development 137: 2507–2517. 20573696 10.1242/dev.049700 PMC2927700

[39] Westerfield M, editor (2000) The zebrafish book. A guide for the laboratory use of Zebrafish (*Danio Rerio*).4th ed.ed: University of Oregon Press, Eugene.

[40] Thisse C, Thisse B (2008) High-resolution in situ hybridization to whole-mount zebrafish embryos. Nat Protoc 3: 59–69. 18193022 10.1038/nprot.2007.514

[41] Smith A, Zhang J, Guay D, Quint E, Johnson A, et al (2008) Gene expression analysis on sections of zebrafish regenerating fins reveals limitations in the whole-mount in situ hybridization method. Dev Dyn 237: 417–425. 18163531 10.1002/dvdy.21417

[42] Akimenko MA, Ekker M, Wegner J, Lin W, Westerfield M (1994) Combinatorial expression of three zebrafish genes related to *distal-less*: part of a homeobox gene code for the head. J Neurosci 14: 3475–3486. 7911517 10.1523/JNEUROSCI.14-06-03475.1994 PMC6576961

[43] Smith A, Avaron F, Guay D, Padhi BK, Akimenko MA (2006) Inhibition of BMP signaling during zebrafish fin regeneration disrupts fin growth and scleroblasts differentiation and function. Dev Biol 299: 438–454. 16959242 10.1016/j.ydbio.2006.08.016

[44] Sleptsova-Friedrich I, Li Y, Emelyanov A, Ekker M, Korzh V, et al (2001) *fgfr3* and regionalization of anterior neural tube in zebrafish. Mech Dev 102: 213–217. 11287195 10.1016/s0925-4773(01)00280-5

[45] Akimenko MA, Johnson SL, Westerfield M, Ekker M (1995) Differential induction of four *msx* homeobox genes during fin development and regeneration in zebrafish. Development 121: 347–357. 7768177 10.1242/dev.121.2.347

[46] Zhang J, Wagh P, Guay D, Sanchez-Pulido L, Padhi BK, et al (2010) Loss of fish actinotrichia proteins and the fin-to-limb transition. Nature 466: 234–237. 20574421 10.1038/nature09137

[47] Avaron F, Hoffman L, Guay D, Akimenko MA (2006) Characterization of two new zebrafish members of the hedgehog family: atypical expression of a zebrafish indian hedgehog gene in skeletal elements of both endochondral and dermal origins. Dev Dyn 235: 478–489. 16292774 10.1002/dvdy.20619

[48] Finckbeiner S, Ko PJ, Carrington B, Sood R, Gross K, et al (2011) Transient knockdown and overexpression reveal a developmental role for the zebrafish *enosf1b* gene. Cell Biosci 1: 32. 21943404 10.1186/2045-3701-1-32 PMC3197473

[49] Verreijdt L, Debiais-Thibaud M, Borday-Birraux V, Van der Heyden C, Sire JY, et al (2006) Expression of the dlx gene family during formation of the cranial bones in the zebrafish (*Danio rerio*): differential involvement in the visceral skeleton and braincase. Dev Dyn 235: 1371–1389. 16534783 10.1002/dvdy.20734

[50] Qiu M, Bulfone A, Ghattas I, Meneses JJ, Christensen L, et al (1997) Role of the *Dlx* homeobox genes in proximodistal patterning of the branchial arches: mutations of *Dlx-1, Dlx-2*, and *Dlx-1* and *-2* alter morphogenesis of proximal skeletal and soft tissue structures derived from the first and second arches. Dev Biol 185: 165–184. 9187081 10.1006/dbio.1997.8556

[51] Qiu M, Bulfone A, Martinez S, Meneses JJ, Shimamura K, et al (1995) Null mutation of Dlx-2 results in abnormal morphogenesis of proximal first and second branchial arch derivatives and abnormal differentiation in the forebrain. Genes Dev 9: 2523–2538. 7590232 10.1101/gad.9.20.2523

[52] Depew MJ, Liu JK, Long JE, Presley R, Meneses JJ, et al (1999) Dlx5 regulates regional development of the branchial arches and sensory capsules. Development 126: 3831–3846. 10433912 10.1242/dev.126.17.3831

[53] Li N, Felber K, Elks P, Croucher P, Roehl HH (2009) Tracking gene expression during zebrafish osteoblast differentiation. Dev Dyn 238: 459–466. 19161246 10.1002/dvdy.21838

[54] Padhi BK, Joly L, Tellis P, Smith A, Nanjappa P, et al (2004) Screen for genes differentially expressed during regeneration of the zebrafish caudal fin. Dev Dyn 231: 527–541. 15376328 10.1002/dvdy.20153

[55] Choo BG, Kondrichin I, Parinov S, Emelyanov A, Go W, et al (2006) Zebrafish transgenic Enhancer TRAP line database (ZETRAP). BMC Dev Biol 6: 5. 16478534 10.1186/1471-213X-6-5 PMC1386650

[56] Bendall AJ, Hu G, Levi G, Abate-Shen C (2003) Dlx5 regulates chondrocyte differentiation at multiple stages. Int J Dev Biol 47: 335–344. 12895028

[57] Zhu H, Bendall AJ (2009) Dlx5 Is a cell autonomous regulator of chondrocyte hypertrophy in mice and functionally substitutes for Dlx6 during endochondral ossification. PLoS One 4: e8097. 19956613 10.1371/journal.pone.0008097 PMC2779492

[58] Esterberg R, Fritz A (2009) *dlx3b/4b* are required for the formation of the preplacodal region and otic placode through local modulation of BMP activity. Dev Biol 325: 189–199. 19007769 10.1016/j.ydbio.2008.10.017 PMC2674874

[59] Depew MJ, Simpson CA, Morasso M, Rubenstein JL (2005) Reassessing the *Dlx* code: the genetic regulation of branchial arch skeletal pattern and development. J Anat 207: 501–561. 16313391 10.1111/j.1469-7580.2005.00487.x PMC1571560

[60] Yang L, Zhang H, Hu G, Wang H, Abate-Shen C, et al (1998) An early phase of embryonic *Dlx5* expression defines the rostral boundary of the neural plate. J Neurosci 18: 8322–8330. 9763476 10.1523/JNEUROSCI.18-20-08322.1998 PMC6792835

[61] Dane PJ, Tucker JB (1985) Modulation of epidermal cell shaping and extracellular matrix during caudal fin morphogenesis in the zebra fish *Brachydanio rerio* . J Embryol Exp Morphol 87: 145–161. 4031750

[62] Lowery LA, Sive H (2004) Strategies of vertebrate neurulation and a re-evaluation of teleost neural tube formation. Mech Dev 121: 1189–1197. 15327780 10.1016/j.mod.2004.04.022

[63] Harrington MJ, Chalasani K, Brewster R (2010) Cellular mechanisms of posterior neural tube morphogenesis in the zebrafish. Dev Dyn 239: 747–762. 20077475 10.1002/dvdy.22184

[64] Coates MI (1994) The origin of vertebrate limbs. Dev Suppl: 169–180. 7579518

[65] Mabee PM, Crotwell PL, Bird NC, Burke AC (2002) Evolution of median fin modules in the axial skeleton of fishes. J Exp Zool 294: 77–90. 12210109 10.1002/jez.10076

[66] Tanaka M, Onimaru K (2012) Acquisition of the paired fins: a view from the sequential evolution of the lateral plate mesoderm. Evol Dev 14: 412–420. 22947314 10.1111/j.1525-142X.2012.00561.x

[67] Tulenko FJ, McCauley DW, Mackenzie EL, Mazan S, Kuratani S, et al (2013) Body wall development in lamprey and a new perspective on the origin of vertebrate paired fins. Proc Natl Acad Sci U S A 110: 11899–11904. 23818600 10.1073/pnas.1304210110 PMC3718130

[68] Yonei-Tamura S, Abe G, Tanaka Y, Anno H, Noro M, et al (2008) Competent stripes for diverse positions of limbs/fins in gnathostome embryos. Evol Dev 10: 737–745. 19021745 10.1111/j.1525-142X.2008.00288.x

[69] Johanson Z (2010) Evolution of paired fins and the lateral somitic frontier. J Exp Zool B Mol Dev Evol 314: 347–352. 20535770 10.1002/jez.b.21343

[70] van Eeden FJ, Granato M, Schach U, Brand M, Furutani-Seiki M, et al (1996) Genetic analysis of fin formation in the zebrafish, *Danio rerio* . Development 123: 255–262. 9007245 10.1242/dev.123.1.255

[71] Matsuoka T, Ahlberg PE, Kessaris N, Iannarelli P, Dennehy U, et al (2005) Neural crest origins of the neck and shoulder. Nature 436: 347–355. 16034409 10.1038/nature03837 PMC1352163

[72] Shearman RM (2005) Growth of the pectoral girdle of the Leopard frog, *Rana pipiens* (*Anura: Ranidae*). J Morphol 264: 94–104. 15744727 10.1002/jmor.10322

[73] Kague E, Gallagher M, Burke S, Parsons M, Franz-Odendaal T, et al (2012) Skeletogenic fate of zebrafish cranial and trunk neural crest. PLoS One 7: e47394. 23155370 10.1371/journal.pone.0047394 PMC3498280

[74] Grandel H, Lun K, Rauch GJ, Rhinn M, Piotrowski T, et al (2002) Retinoic acid signalling in the zebrafish embryo is necessary during pre-segmentation stages to pattern the anterior-posterior axis of the CNS and to induce a pectoral fin bud. Development 129: 2851–2865. 12050134 10.1242/dev.129.12.2851

[75] Ahn DG, Kourakis MJ, Rohde LA, Silver LM, Ho RK (2002) T-box gene *tbx5* is essential for formation of the pectoral limb bud. Nature 417: 754–758. 12066188 10.1038/nature00814

[76] Gibert Y, Gajewski A, Meyer A, Begemann G (2006) Induction and prepatterning of the zebrafish pectoral fin bud requires axial retinoic acid signaling. Development 133: 2649–2659. 16774994 10.1242/dev.02438

[77] He X, Yan YL, Eberhart JK, Herpin A, Wagner TU, et al (2011) *miR-196* regulates axial patterning and pectoral appendage initiation. Dev Biol 357: 463–477. 21787766 10.1016/j.ydbio.2011.07.014 PMC3164755

[78] Crotwell PL, Mabee PM (2007) Gene expression patterns underlying proximal-distal skeletal segmentation in late-stage zebrafish, *Danio rerio* . Dev Dyn 236: 3111–3128. 17948314 10.1002/dvdy.21352

[79] Ferrari D, Kosher RA (2006) Expression of *Dlx5* and *Dlx6* during specification of the elbow joint. Int J Dev Biol 50: 709–713. 17051482 10.1387/ijdb.062180df

[80] Crotwell PL, Clark TG, Mabee PM (2001) *Gdf5* is expressed in the developing skeleton of median fins of late-stage zebrafish, *Danio rerio* . Dev Genes Evol 211: 555–558. 11862461 10.1007/s00427-001-0186-z

