# Supplementary figures and images for: The dlx5a/dlx6a Genes Play Essential Roles in the Early Development of Zebrafish Median Fin and Pectoral Structures
Source: PLoS One. 2014 May 23;9(5):e98505. doi: 10.1371/journal.pone.0098505 (PMC4032342; doi:10.1371/journal.pone.0098505)

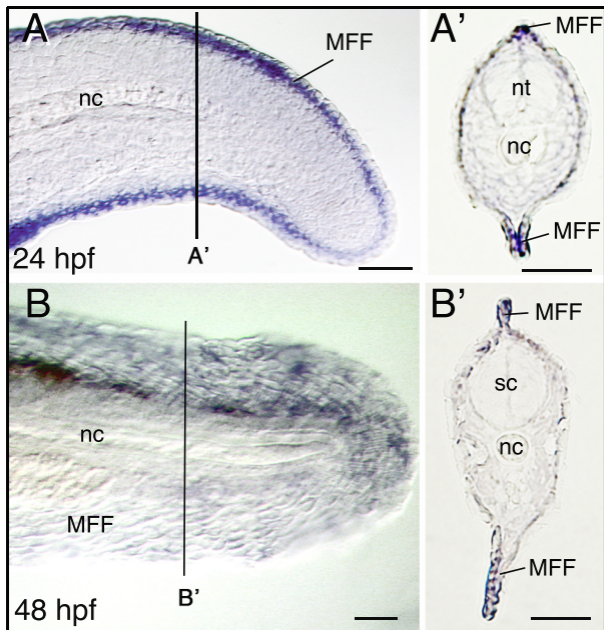

Fig. S1

Supplement: Figure S1 — Expression patterns of dlx5a during zebrafish median fin development. Whole mount in situ hybridization for dlx5a on lateral view of the posterior axis of 24 hpf (A) and 48 hpf (B) zebrafish embryos and on 10 µm parasagittal frozen sections (A′, B′) at the level indicated in (A, B). At 24 and 48 hpf, dlx5a expression is limited to apical ectodermal cells of the median fin fold (MFF). nc, notochord; nt, neural tube; sc, spinal cord. Scale bars 50 µm. (PDF) [file pone.0098505.s001.pdf]

*dlx5a*

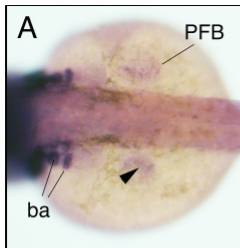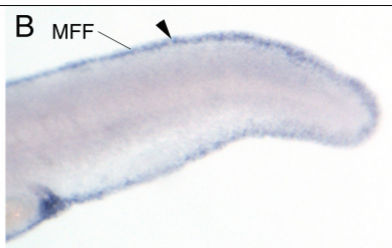

*dlx6a*

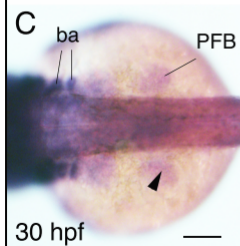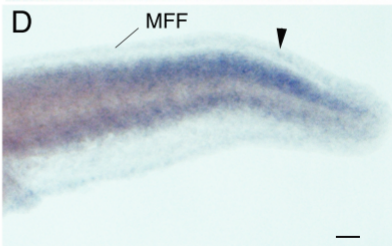

Fig. S2

Supplement: Figure S2 — Comparison of dlx5a and dlx6a expression in the developing zebrafish fins. Whole mount in situ hybridization for dlx5a (A, B) and dlx6a (C, D) in the pectoral fin bud (PFB) (A, C) and in the median fin fold (MFF) (B, D) of 30 hpf embryos. Expression of dlx5a is detected in apical ectodermal cells of both pectoral and median developing fins (A, B, black arrowheads). Expression of dlx6a mirrors dlx5a expression, however dlx6a transcripts seem to be present at lower level (C, D, black arrowheads). Scale bars 50 µm. (PDF) [file pone.0098505.s002.pdf]

dorsal view

lateral view

*runx2b*

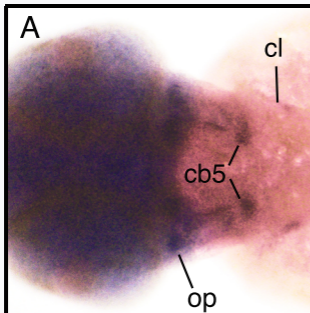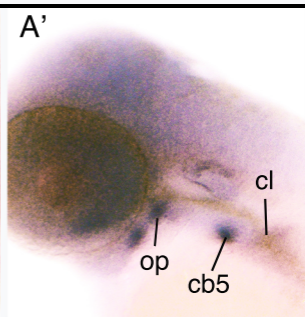

*dlx5a*

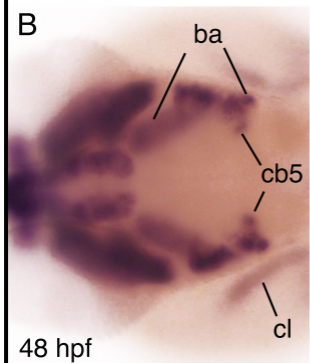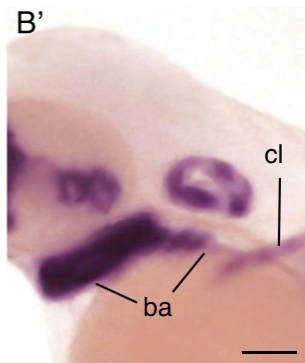

Fig. S3

Supplement: Figure S3 — The opercle, the ceratobranchial-5 bone and the cleithrum all develop in a dlx5a -positive context. Dorsal and lateral views of whole mount in situ hybridization for runx2b (A, A′) and dlx5a (B, B′) in the anterior region of 48 hpf zebrafish embryos. Expression of runx2b reveals the opercle (op), the ceratobranchial-5 bone (cb5) and the cleithrum (cl) (A, A′), structures which differentiate at early stage of zebrafish development. The dlx5a expression analysis at equivalent stage shows that the three bones develop in dlx5a-positive domains, explaining the loss of runx2b expression in dlx5a/6a morphants at the pectoral and craniofacial levels shown in Fig. 5 D (black and blue asterisks). ba, branchial arches. Scale bars 100 µm. (PDF) [file pone.0098505.s003.pdf]

*and1*

CT

A

AP

B

*dlx5a/6a* MO

24 hpf

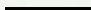

Fig. S4

Supplement: Figure S4 — Expression of and1 in the median fin fold of control and dlx5a/6a morphant embryos. Ventral view at the posterior level of whole mount in situ hybridization for and1 in 24 hpf controls (A) and dlx5a/6a morphants (B). The ventral view reveals that and1 transcripts are not expressed in the apical ectodermal cells of the median fin fold (medial line, AP), but in ectodermal cells adjacent to the AP. Scale bar shown in B for the two panels 50 µm. (PDF) [file pone.0098505.s004.pdf]

CT

*dlx5a/6a* MO

BrDU

A

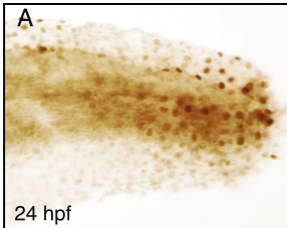

24 hpf

A'

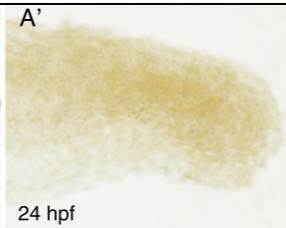

24 hpf

TUNEL

B

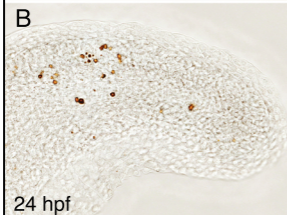

24 hpf

B'

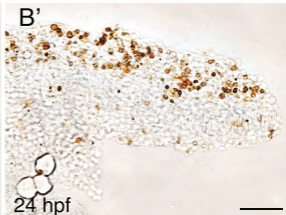

24 hpf

Fig. S5

Supplement: Figure S5 — The median fin fold defects in dlx5a/6a morphants are associated with altered cell proliferation and apoptosis. Lateral view at the posterior axis of BrdU (A, A′) and TUNEL (B, B′) assays on control (A–B) and dlx5a/6a morphant (A′–B′) embryos at 24 hpf. The BrdU assay shows that controls present proliferating cells in the median fin fold whereas no BrdU-positive cells are observed in the morphants. In parallel, the morphants show a high increase of apoptotic cells in the MFF compared to controls (B–B′). Scale bar shown in B′ for all panels 20 µm. (PDF) [file pone.0098505.s005.pdf]

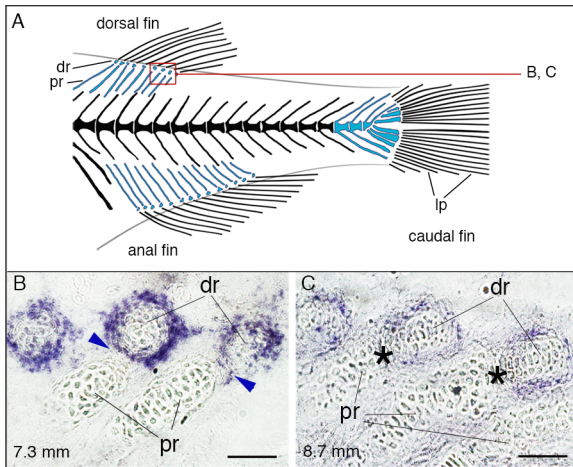

Fig. S6

Supplement: Figure S6 — Expression of dlx5a in fin skeletal components of late-stage zebrafish larvae. (A) Overview of the posterior axial skeleton of a one-month-old zebrafish. Endoskeletal fin supports are colored blue and red square indicates the structures analyzed in (B–C). (B, C) Whole mount in situ hybridization for dlx5a on 10 µm parasagittal frozen sections of 7.3 mm and 8.7 mm late-stage zebrafish. As seen in Fig. 8 C in 6.6 mm larvae, dlx5a expression is still detected in cells surrounding the distal radials (dr) in 7.3 mm larvae, including in the zone of segmentation (ZS) (B, blue arrowheads) when the radial segmentation is almost completed. Later, after segmentation (8.7 mm), dlx5a expression is maintained but decreases in cells surrounding the distal radials and is no longer detected in the ZS (black asterisks) (C). dr, distal radials; lp, lepidotrichia; pr, proximal radials. Scale bars B–C 10 µm. (PDF) [file pone.0098505.s006.pdf]
